# Ancient diversity of *Triticum aestivum* subspecies as source of novel loci for bread wheat improvement

**DOI:** 10.3389/fpls.2025.1536991

**Published:** 2025-04-09

**Authors:** Delfina Barabaschi, Andrea Volante, Primetta Faccioli, Alice Povesi, Ivana Tagliaferri, Elisabetta Mazzucotelli, Luigi Cattivelli

**Affiliations:** ^1^ Council for Agricultural Research and Economics (CREA) - Research Centre for Genomics and Bioinformatics, Fiorenzuola d’Arda, Italy; ^2^ Council for Agricultural Research and Economics (CREA) - Research Centre for Vegetable and Ornamental Crops, Sanremo, Italy

**Keywords:** wheat, hexaploid subspecies, variability, genome-wide association study, favorable allele

## Abstract

Ancient subspecies of hexaploid wheat, not yet subjected to intensive selection, harbor potentially valuable alternative genetic variability for the genetic improvement of modern cultivated bread wheat. To investigate these hitherto unexplored resources, we established a panel, currently unique, consisting of 190 accessions of *Triticum aestivum* belonging to five different neglected subspecies, *compactum*, *sphaerococcum*, *macha*, *spelta*, and *vavilovii*, with few *aestivum* references. The panel was genotyped through the iSelect Illumina arrays (20K and 25K) and phenotyped for 25 traits related to phenology, morphology, yield, and physiology for 4 years under field conditions. We found wide variability for all traits analyzed, both within and among subspecies, demonstrating the richness contained therein. Through a genome-wide association study (GWAS), we identified a total of 126 marker–trait associations (MTAs), including 4 for years, 58 for morphological traits, 39 related to yield, and 25 for physiological traits, some of them confirming loci previously published and others being novel. Fourteen MTAs were associated with multiple traits. Among them, one on chromosome 2D at 360.2 Mb was associated with spike density, length, and shape, and thus is of particular interest because it may underlie the compactum (*C*) gene, until now considered difficult to clone because of its centromeric position. The physical distance defined by this MTA is considerably smaller (1.7 Mb) than what is reported so far in the literature, paving the way toward physical mapping of the *C* gene. A potential candidate gene has been identified for the trait grain number per spike. This is *TraesCS6A03G0476500*, coding for a monosaccharide-sensing protein 2, located on chromosome 6A at 233 Mb and identified through an MTA that segregates exclusively in *compactum* accessions. The results obtained confirm the remarkable potential present in the panel of wheat subspecies analyzed in this study, which, being characterized by a very short linkage disequilibrium (LD) decay, allowed the definition of rather narrow ranges around key traits, such as those related to yield, providing new perspectives on transferring genes across subspecies for wheat improvement.

## 1 Introduction

Wheat (*Triticum* ssp.) has been the world’s most widely cultivated food crop since the beginning of agriculture. Nowadays, there is an increasing global demand for novel varieties with improved crop yield and disease resistance to be suitable to divergent climatic conditions, and with innovative quality traits. In this context, the exploitation of the genetic resources plays a key role to support the genetic improvement of crop plants acting as sources of new favorable alleles to be introduced in the gene pool of elite cultivars. For this purpose, over 80 distinct germplasm collections holding an estimated total of more than 800,000 accessions are available (Wheat | Crops | Resources | CGIAR Genebank Platform).

Common or bread wheat (*Triticum aestivum* L., hexaploid genome AABBDD) is one of the most widely grown crops worldwide and the staple food for most countries (Tadesse et al., 2019). Within hexaploid wheat primary gene pool, the ancient subspecies (which include landraces, wilds, and old cultivars) can be considered “exotic” genetic materials, and hence valuable sources of new, potentially favorable alleles. Wheat ancient subspecies (ssp.) may represent a reservoir of alternative variability that have been left behind during the processes of range extension and modern breeding under intensive agricultural management (Fu, 2015). The hexaploid subspecies differ quite well from each other in spike architecture (spike shape, density, and the seed shape), which is mainly controlled by three major genes (Fan et al., 2019): the spelt factor *q* (speltoid spike: a spear-shaped spike with an elongated, brittle rachis, and non-free-threshing grains) and its dominant allele *Q* (square-headed, free-threshing grain and tough rachis) on chromosome arm 5AL (Simons et al., 2006); the gene for ear compactness on chromosome arm 2DL (*C/c*: compact spike/non-compact spike) with pleiotropic effects on grain size, shape, and number per spike (Johnson et al., 2008); and finally the sphaerococcum factor (*S1/s1*: non-spherical/spherical grains), which controls seed shape, on 3D (Faris et al., 2014).

In the present work, a germplasm collection of 190 hexaploid wheat accessions (hereafter called “*Ta*-ssp panel”) has been set up, originated worldwide and hence adapted to different agro-climatic zones. Besides few cultivars of *T. aestivum* ssp. *aestivum* acting as references, the panel includes a significant representation of four *T. aestivum* subspecies: *compactum*, *sphaerococcum*, *macha*, and *spelta*, plus one *vavilovii*.

About half of the collection is composed of spelt forms (*macha*, *spelta*, and *vavilovii*) characterized by intrinsic and high polymorphism due to several independent hybridizations involving different tetraploid forms (Kuckuck, 1964), although *T. dicoccum* seems to be the most favored. *Macha*, much more widely distributed in prehistoric ages, has nowadays a very scarce distribution; in the past, it has been proposed as the primitive hexaploid of other spelt wheat (Dorofeev, 1972). It is quite tall and characterized by laterally compressed wide fragile ears, awned or awnless; it is endemic to Georgia (Bedoshvili et al., 2021). *Spelta* has long lax fragile ears, awned or awnless, with tightly covered grains; it has a long cultivation tradition in southwest Germany, Austrian Alpine valleys, Switzerland, and a few other remote areas of Europe (Grausgruber, 2018). *Vavilovii*, probably a variant of *spelta*, is cultivated only in Armenia and carries branched ears, characterized by a lengthening of the spikelet axis and short awns (Dorofeev et al., 1979).

Like *aestivum*, *sphaerococcum* and *compactum* are both free-threshing wheat; indeed, all carry the dominant Q allele. *Sphaerococcum*, also known as Indian dwarf wheat, was widespread in prehistory and before the Green Revolution. It is characterized by short dense awned or awnless ears and small near-hemispherical grains with a high protein content; it is endemic to southern Pakistan and northwestern India (Szczepanek et al., 2020). *Compactum*, also known as club wheat, is characterized by short ears, uniformly dense, and oblong or oval, which can be awned or awnless. Cultivars of *compactum* are still commercially competitive in the U.S. Pacific Northwest, while their distribution is limited to a few areas in Europe, Turkey, and Australia. They also exhibit drought resistance and earliness (Johnson et al., 2008).

*Spelta* is currently the subspecies that attracts the most attention and interest, despite showing brittle rachis and grains tightly surrounded by tenacious glumes. Recently, the spelt market increased globally, owing to the demand for ancient wheat being less subjected to modern breeding (Chrpová et al., 2021). Its origin and relationship with the *aestivum* subspecies are still debated; indeed, these two subspecies are evolutionary very closely related, but still significantly divergent at the sequence level (Liu et al., 2018). In addition, *spelta* is differentiated into two geographical groups, coming from Europe and Asia. Hyun et al. (2020) suggested a polyphylesis for the A and B genome for the European and Asian spelt wheat, and a common ancestor for the D genome, with the latter monophylesis being already suggested by Dvorak et al. (2012) for all subspecies of *T. aestivum*.

European spelt wheat could be derived from an independent hybridization of domesticated emmer wheat (*T. turgidum* ssp. *dicoccum*) with *Ae. tauschii* (McFadden and Sears, 1946; Kerber and Rowland, 1974), or with a free-threshing hexaploid wheat (Yan et al., 2003; Blatter et al., 2004), while Asian spelt could have evolved from the ancestral *T. aestivum* ssp. *spelta*; the latter, together with *T. aestivum* ssp. *Aestivum*, could be derived from primitive hexaploid wheat. Very recently, Wang et al. (2024) refined an evolutionary model at least for European spelt by proposing interspecific hybridization and gene flow between primitive hexaploid wheat and domesticated emmer wheat. The evolutionary relationships among the above-mentioned *T. aestivum* subspecies are still unclear. The naked, compact, spherical-seeded forms appear to have arisen after hybridization between *T. dicoccum* and *Ae. tauschii* as the result of single mutations. Indeed, *compactum*, the origin of which has been discussed in the past, could be raised from a mutation at the C locus in *aestivum* (Johnson et al., 2008). Phylogenetic differences were also confirmed between *macha* and *aestivum* as well as between *macha* and *spelta* (Cao et al., 2000).

Such a diversified material offers unique opportunities to enrich the available bread wheat germplasm with new valuable alleles and to increase the diversity of the existing materials; a detailed genetic and agro-morphological characterization of a *Ta*-ssp panel is of paramount importance to provide information on this hitherto underexploited genetic resource and to set up focused approaches for breeding programs. More than 20K SNPs (single-nucleotide polymorphisms) were used in this study to describe the genetic structure of the *Ta*-ssp panel and to investigate the diversity between and within the subspecies. We undertook a GWAS for several phenological, morphological, physiological, and yield-related traits, and a remarkable set of novel MTAs (marker–trait associations), besides some already known, have been detected, providing the starting point for mining candidate genes underlying the traits of interest. This study represents the first comprehensive attempt to widen the knowledge of existing wheat hexaploid germplasm through the characterization of ancient subspecies.

## 2 Material and methods

### 2.1 Assembly and composition of the subspecies panel

A panel of 190 hexaploid wheat accessions (hereafter defined as the “*Ta*-ssp panel”), mostly obtained from the National Small Grains Collection (USDA-ARS) (Search Accessions GRIN-Global - ars-grin.gov), was evaluated in this work. The original seeds were used to produce single seed descent through one cycle of self-fertilization during field trials in Fiorenzuola d’Arda (Italy) in the 2017–2018 growing season.

The accessions belong to six different subspecies (ssp.) of *T. aestivum*: 15 *T. a*. ssp. *aestivum*, 58 *T. a.* ssp. *compactum*, 26 *T. a.* ssp. *sphaerococcum*, 24 *T. a.* ssp. *macha*, 66 *T. a*. ssp. *spelta*, and 1 *T. a.* ssp. *vavilovii*. This panel includes cultivars, breeding materials, landraces, and wilds (the latter are all *macha*, plus the *vavilovii*). The *aestivum* accessions present in the panel are representative of the genetic diversity of the modern cultivars and include two genotypes for which reference-quality genome is available: Chinese spring and Arina (Walkowiak et al., 2020, https://galaxy-web.ipk-gatersleben.de/libraries/folders/F1cd8e2f6b131e891/page/1).

The improvement status (according to GRIN) was reported when available. Most of the cultivars belong to *aestivum* or *compactum*, while 60% of *spelta* accessions are landraces. The genotypes originated from five continents and 40 different countries, with the most represented being USA (26), Germany (11), India (10), and Austria (10). Detailed information for each accession is given in **Table 1** and **Supplementary Table S1**.

**Table 1:** Composition of the *Ta*-ssp panel: number of accession/subspecies, distribution by countries and continents, and allele combination of three major genes controlling spike architecture (the spelt factor, the gene for ear compactness, and the sphaerococcum factor).

*Triticum aestivum* subspecies*	No. of accessions	Common name	Origin (number of countries/continents)	Tough rachis and free-threshing (chr.5A)^°^	Compact ear (chr.2D)^§^	Spherical grain (chr.3D)^#^
*Aestivum*	15	Bread wheat	9/4	QQ	cc	SS
C*ompactum*	58	Club wheat	23/5	QQ	CC	SS
*Sphaerococcum*	26	Indian dwarf	11/3	QQ	cc	ss
*Macha*	24	–	11/3	qq	CC	SS
*Spelta*	66	Spelt wheat	23/4	qq	cc	SS
*Vavilovii*	1	–	1/1	qq	cc	SS

* *Aestivum: Triticum aestivum* ssp. *aestivum; Compactum: T. a.* ssp. *compactum; Sphaerococcum: T. a.* ssp. *sphaerococcum; Macha: T. a.* ssp. *macha; Spelta: T. a.* ssp. *spelta; Vavilovii: T. a.* ssp. *vavilovii*.

° Q: square-headed/free-threshing grain/tough rachis; q: speltoid/non-free-threshing grain/brittle rachis.

§ C: compact spike; c: non-compact spike.

# S: non-sphaerococcum grain; s: sphaerococcum grain.

### 2.2 Field trials, phenotypic trait evaluation, and statistical analysis

A field trial was set up at the experimental farm in Fiorenzuola d’Arda, Italy (44˚ 55′22″ N, 9˚ 54′36″ E) under rainfed conditions during four crop seasons: 2017–2018, 2018–2019, 2020–2021, and 2021–2022 (hereafter called only 2018, 2019, 2021, and 2022). Fields were managed for pests and diseases, and with fertilizer applications following the local agronomic practices. Genotypes were tested in a randomized complete block design in a single row (1 m long using a sowing density of 25 seeds/m) and three (in 2019) or two (in 2018, 2021, and 2022) replications per genotype. In the first year (2018), only 151 accessions out of 190 of the full *Ta*-ssp panel were available and hence evaluated. A total of 25 agronomic traits were evaluated across years and accessions (for details, see **Supplementary Table S2**).

Days to heading (DTH) was calculated as the number of days from 1 January to when spikes appeared in at least 50% of the plants. Regarding plant morphology, 10 traits were considered at suitable developmental stages. At physiological maturity, two traits were recorded: plant height (PH, cm), measured from the soil level to the tip of the spike (excluding awns), and the angle (FLANG) and elasticity (FLE) of flag leaf through a visual assessment. FLANG was scored using the following scale: 1 for erect leaves (0–30°), 2 for intermediate or horizontal leaves (31–90°), 3 for semi-pendant leaves (91–130°), and 4 for pendant leaves (131–180°), while FLE, defined as the response to a moderate pressure applied with a finger, was evaluated within a 1–4 range of elasticity: 1, very poor; 2, poor; 3, good; 4, very good. Flag leaf size was estimated by length (FLL, cm), width (FLW, cm), and area (FLA, cm^2^) at the milky ripe stage, on five randomly selected plants from the main tillers of each row. The leaf samples were collected, pasted on a sheet of paper, and immediately measured for FLL from leaf base to leaf tip and FLW at the widest position of flag leaf lamina with a ruler with an accuracy of 1 mm. FLA was determined from digital images acquired by an STD 10000XL scanner (Epson, Japan) and then analyzed with the package “LeafArea”, employed to run ImageJ software within R.

Awn presence (AP), glume color (GLC), and spike shape (SS) were determined post-harvesting, on five main tiller spikes of each accession. AP was assessed following this scale: 0 = awnless; 3 = awnletted (short awns); 7 = awned (conspicuous awns). GLC, observed on the outer glume, was measured within a 1–3 range: 1 = white; 2 = red to brown; 3 = purple to black; SS was assessed on a visual 1–9 scale: 1 = tapering; 3 = parallel side; 5 = semi-clavate; 7 = clavate; 9 = fusiform. Grain color (GC) was evaluated after threshing within a 1–3 scale: 1 = white; 2 = red and brown; white, 3 = purple.

Eight yield-related traits were recorded onto the same 5 spikes used for the above measurements. Spike length (SL, cm) was measured from the base of the rachis to the tip of the uppermost spikelet without awns. Then, spike weight (SW), spikelet number per spike (SNS), and among them those fertile (FSNS) were evaluated. Spike density (SD) was studied in two ways: (i) on a 1–9 scale: 1 = very lax, 3 = lax, 5 = intermediate, 7 = dense, 9 = very dense; and (ii) as dividing SNS by SL, multiplied *10 (SD^). The number of grains per spike (GNS) and their total weight (GWS) were also assessed, followed by thousand grain weight (TGW) obtained as an average weight of 100 grains × 10 (2 replicates).

Physiological traits were also recorded. Leaf chlorophyll (CHL) and epidermal flavonoids (FLV) were measured at 10 days after heading using Dualex 4 Scientific (Dx4) (FORCE‐A, Orsay, France), on five randomly selected plants from the main tillers of each row. Three readings were taken from both sides (superior and inferior) of each leaf: CHLs and CHLi, and FLVs and FLVi. The simultaneous non-destructive assessment of these two physiological traits allowed one to calculate the nitrogen balance index (NBI) as the ratio between CHL and FLA content, an indicator of C/N allocation balance (Cartelat et al., 2005).

In 2018, GC, SW, FSNS, SD, SD^, and GWS were not recorded since the panel was not yet complete; the evaluation of TGW was added only in the last 2 years (2021 and 2022) while FLANG and FLE were not recorded in 2022 due to lodging caused at the time of the survey.

All statistical analyses were performed with R packages. The phenotypic data were analyzed by fitting linear models using the lmer function of the lme4 package (Bates et al., 2015). Best Linear Unbiased Predictors (BLUPs) of each trait for each accession were calculated using the ranef function. The model fitted the data considering genotype, year, and genotype × year interaction as random factors. The model was formalized as follows:


Yijk= µ + Gi + Yj + Rk(j) + GEij + eijk

where *Y_ijk_* is the phenotypic value of the trait of interest of genotype *i* in year *j* and replication *k*, *µ* is the overall mean, *G_i_* is the effect of genotype *i*, *Y_j_* is the effect of the year *j*, *R_k(j)_* is the effect of replication *k* nested in year *j*, *GY_ij_* is the *G* × *Y* interaction between genotype *i* and year *j*, and *e_ijk_* is the residual effect associated with genotype *i* in year *j* and replication *k*.

An analysis of variance (ANOVA) was performed on BLUPs, using genotypes, years, and subspecies as factors. Broad-sense heritability (H^2^) was estimated from variance components across years according to the following equation (Nyquist, 1991):


$$H2=[\frac{Vg}{Vg+\frac{Ve}{r}}]\;$$

where $Vg=\frac{\left[MS(G)-MS(E)\right]}{r}\;\;Ve=MS(E)$ In these expressions, *r* is the number of years, and MS(G) and MS(E) are the mean sums of squares for genotype and residual error obtained from analysis of variance.

Pearson pairwise correlation among agronomic traits was computed using the BLUPs over environments with the R package corrplot (Taiyun and Simko, 2024).

### 2.3 DNA extraction and genotyping for known genes

Genomic DNA was isolated from plant tissue samples obtained at the three-leaf stage, according to a standard CTAB-based protocol (Stein et al., 2001). DNA integrity and quantity were checked and confirmed. Markers (simple sequence repeat—SSR, sequence-tagged site—STS, or cleaved amplified polymorphic sequences—CAPS) for specific loci, from previously published studies, affecting plant height, plant phenology, vernalization, and genes for quality traits such as hardness (*Pinb-D1*), grain protein content (*Gcp-B1*), and grain weight were analyzed by polymerase chain reaction (PCR). Detailed information for each marker, protocol, and reference is present in **Supplementary Table S3**.

### 2.4 Genotyping with high-throughput array and *in silico* physical mapping

Genotyping of the 190 wheat accessions was conducted at Trait Genetics, Gatersleben (Germany, https://sgs-institut-fresenius.de/en/health-nutrition/traitgenetics), by using the 20K and later 25K iSelect arrays (Illumina Inc, San Diego, USA). The 20K array contains a total of 17,267 SNPs: a combination of a subset of high-quality and predominantly haplotype-specific markers from the 90K iSelect array (Wang et al., 2014), plus a small set of candidate genes for major phenology traits such as *Ppd* and *Rht* and some quality traits from the 820K Axiom array (Winfield et al., 2016). The 25K array contains most of the markers from the previous 20K array plus additional SNPs from 135K Affymetrix wheat array and candidate genes. Samples were analyzed on Infinium ultra-HD chips carrying 24 samples each and allele calling was performed. Combining the two arrays, a total of 24,369 SNPs were available for the detection of genetic diversity. Markers with more than 10% of missing values, a minor allele frequency (MAF) lower than 5%, and individuals with more than 5% missing SNP calls were filtered out. After this QC check, 20,729 high-quality SNP markers remained for all the 190 lines.

Flanking sequences of each SNP (available in Gogna et al., 2022) were mapped against the reference wheat genome of Chinese spring produced by the International Wheat Genome Sequencing Consortium (IWGSC RefSeq v2.1; https://urgi.versailles.inra.fr/blast_iwgsc/, IWGSC, 2018) to get their putative physical location. For the blastn (run locally at https://wheat-urgi.versailles.inra.fr/Seq-Repository/Assemblies), the following parameters were used: an e-value cutoff of e^−30^, an identity higher than or equal to 97%, and a sum of gaps and mismatches no higher than 3 (including the SNP position). In addition, only reads that uniquely map to one of three subgenomes were retained. Among the 20,729 filtered markers, a physical position was obtained for 14,640; 58% of them had an extremely robust hit, showing a full-length alignment with the only exception of the SNP (defined as perfect match). A total of 40 markers were not assigned to any chromosomal position (unknown).

### 2.5 Analysis of population structure

The genetic stratification in the *T. aestivum* subspecies panel was inferred, using the set of physically mapped SNPs (14,680), by three independent methods: neighbor-joining (N-J) clustering method, principal coordinates analysis (PCoA), and a Bayesian model-based analysis.

The N-J tree was developed with MEGA v.7 (Kumar et al., 2016), using a total of 1,000 bootstrap replicates and Jukes–Cantor genetic distance (Simonetti et al., 2022; El-Esawi et al., 2023). The tree file was exported in Newick format to be graphically represented with iTOL version v6 (https://itol.embl.de/.), together with the information relative to different subspecies. A PCoA was performed using PAST v4.03 (Hammer et al., 2001) based on the Hamming distances. The first and second principal coordinates were used to draw the two-dimensional space graph, onto which subspecies attributions were projected.

The Bayesian model-based analysis was achieved using Structure 2.3.4 (Pritchard et al., 2000). This software determines the number of optimal clusters (*K*) in the panel and estimates the membership probability of each genotype in each subpopulation. All analyses were run with the ADMIXTURE model, with correlated allele frequencies, a burn-in period of 10,000 iterations, followed by 20,000 Monte Carlo Markov chain (MCMC) replications from *K*=1 to *K*=10. Five independent runs were generated for each *K*. The results of the analysis were used as input to the Structure Selector tool (Earl and von Holdt, 2011) to predict the best *K*-value based on the Evanno method (Δ*K* method; Evanno et al., 2005). One final run was performed, at the most probable *K*-value, using the same parameters listed above, but with a higher number of burn-in and MCMC iterations (100,000 and 200,000, respectively). Accessions were assigned to subpopulation if the probability of membership was greater than 70% (Volante et al., 2017); the remaining accessions were considered as admixed. The number and composition of the clusters identified were integrated into the results of the N-J analysis and the PCoA for cross-validation.

### 2.6 Analysis of linkage disequilibrium decay

The extent of chromosome-wise linkage disequilibrium (LD) was evaluated by calculating the pairwise LD squared allele frequency correlation coefficient (*r* ^2^) for all intra-chromosomal SNP pair loci with physical positions (14,680) as described in Volante et al. (2021). The computation was done with the R package “LDcorSV v1.3.1” (Mangin et al., 2012) using as covariates the structure membership matrix and the kinship matrix calculated by Tassel v.5 (Bradbury et al., 2007). Afterwards, the *r* ^2^ values were binned in 100-Kb windows and the median value of each distance class was calculated for each of the 21 linkage groups (this was used instead of the mean as LD values are not normally distributed at short and long-distance classes). The resulting values were plotted against physical distance (in Kb) and fitted to a second-degree LOESS curve using an R script (Cleveland, 1979; Marroni et al., 2011). A critical value of 0.2 was set as *r* ^2^ between unlinked loci, and the LD decay rate in the panel was calculated as the physical distance corresponding to the LOESS curve value at the critical 0.2 threshold. LD decay was estimated for each chromosome and for each genome.

### 2.7 Genome-wide association study

The GWAS was performed using mixed linear model (MLM) implemented by Genomic Association and Prediction Integrated Tool (GAPIT) in R (Lipka et al., 2012). A K-PC model was used (Zhao et al., 2007), where kinship information together with the first three principal components (PCs) as covariates were included for GWAS, to further improve statistical power. “Optimal compression and no P3D mode” parameters were used to examine associations between SNP markers and phenotypic data. GWAS was conducted on BLUPs calculated on phenotypic data scored across years for each accession to account for the genotype × year interaction on trait variability.

To identify loci (MTAs) associated with the 25 agronomic traits evaluated in this work, association analysis was performed using the entire set of informative SNPs (20,729).

The *p*-value of the association to the phenotypic trait was computed for each marker. All markers with a threshold of −log_10_ (*p*-value) ≥ 3.5 were assumed to be associated with traits, highlighting those under the significance threshold of 0.05 of false discovery rate (Benjamini and Hochberg, 1995) and those above the Bonferroni threshold, considered highly associated. MTAs were visualized using Manhattan plots for each trait. To determine whether the MTAs detected in this study were in the same position as that of previously reported genes, MTAs, or QTL, the physical locations of the genomic regions were compared. All markers associated with a specific trait were assigned to the same MTA regions when distance between them was less than LD decay of the chromosome. The peak marker was defined as that with the highest −log_10_ (*p*-value) value detected in the region.

High-confidence (HC) genes located next to those MTAs associated with multiple traits were searched in JBrowse-IWGSC RefSeq (https://wheat-urgi.versailles.inra.fr/Tools/JBrowse), taking as interval the extent of LD as calculated for each chromosome on the left and right side of the MTA. The annotation of each HC gene was also retrieved from Wheat URGI repository (https://wheat-urgi.versailles.inrae.fr/Seq-Repository/Annotations) and reported. For the regions deemed most interesting, the expression patterns of HC genes in different organs and developmental stages were retrieved by wheat mRNA transcription data in the wheat expression database (Wheat eFP Browser).

## 3 Results

### 3.1 Phenotypic variation in the *Ta*-ssp panel

A broad variation for all measured traits was detected within and among subspecies. Means, median, minimum, maximum, and quartiles for all traits in each year were calculated for the entire panel (**Supplementary Table S4A**) and minimum, maximum, and mean values were also measured for each subspecies in each year (**Supplementary Table S4B**). The mean values of the 21 non-categorical traits analyzed in at least two growing seasons were also evaluated and reported in **Supplementary Table S4C** to provide a general overview of the different morphologies and behaviors among the subspecies.

The distribution of the traits among the diverse subspecies generally followed the same trend across years (**Supplementary Figure S1**). *Sphaerococcum* is the subspecies that heads the earliest and the one with the lowest plant height and narrowest leaf angle, the one that is least elastic, and the one with the overall smallest leaves (length, width, and area) and the lowest number of seeds and spikelets per spike (total and fertile), as well as the one with the lowest GWS. *Macha* accessions were found to be the most late in heading, the tallest, and the most elastic, and the ones with the highest spikelet number per spike (total and fertile), while *spelta* accessions were characterized by the widest leaf angle, the longest ears, and the highest TGW. The number and weight of seeds per spike as well as spike weight and derived density (SD^) were highest in *compactum*, associated with shorter spike length. The highest chlorophyll content was detected in aestivum subspecies, while the lowest was found in *macha* subspecies (**Supplementary Table S4C**). From these general comparisons, we excluded *vavilovii* since it was being represented by a single accession in the *Ta*-ssp panel.

**Figure 1:**
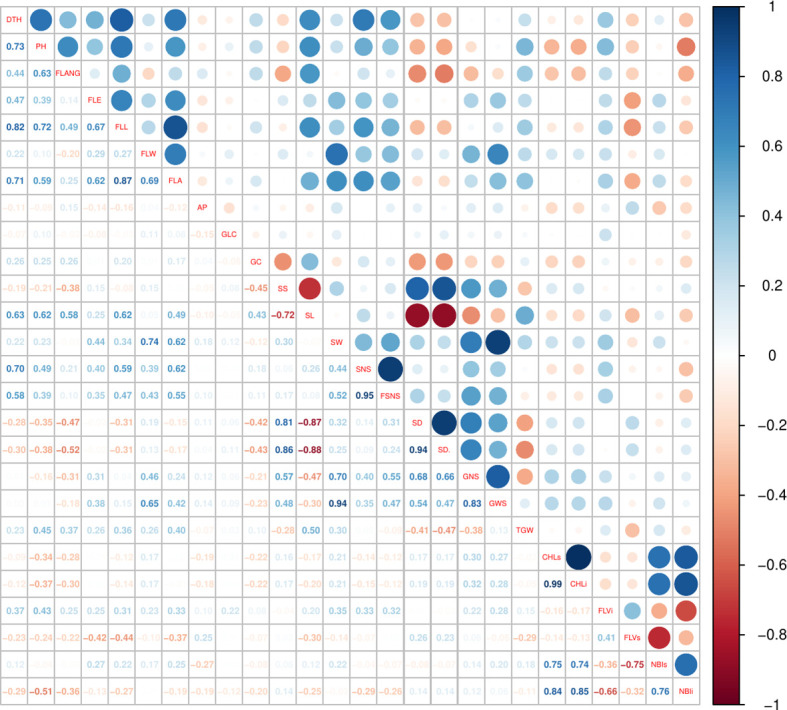
Pearson correlations among the phenotypic traits recorded in the *Ta*-ssp panel.

The traits showing the lowest coefficients of variation (CVs) were FLVs and FLVi, ranging from 7.11% to 11.71% (over the 4 years), followed by CHLs and CHLi. The highest variability (between 42.44% and 51.40%) was observed for SL, except in 2019 since not all subspecies were measured (see the Material and Methods section) (**Table 2**). ANOVA calculated on BLUPs showed that genotypes, subspecies, and year had significant effects on phenotypic variation with the only exception of TGW, for which only year had a strong effect (**Table 2**). Broad-sense heritability (H^2^) values were very high (>85%) for DTH, PH, FLL, SL, GNS, SNS, FSNS, and SD^ and rather low for physiological traits, ranging between 57.6% (NBIi) and 62.0% (CHLi). Pearson analysis was used to highlight significant baseline correlations between different traits (**Figure 1**, **Supplementary Table S5**). Very high positive correlations (above *r* = 0.80) were found between DTH ad FLL (0.82), between SS and SD^ (0.86), between SW and GWS (0.94), and between GNS and GWS (0.83), while SD and SL were strongly negatively correlated (−0.86). Other moderate positive correlations (0.60 ≤ *r* ≤ 0.80) were observed: DTH with FLA, PH, and SL; PH with FLANG, FLL, and SL; and FLE with FLL and FLA.

**Table 2:** Descriptive statistics of all quantitative traits evaluated in 4 years, and ANOVA and heritability on BLUPs.

						ANOVA on BLUPs	
Trait*	Year^a^	Mean	SD^b^	Range	CV (%)^c^	Genotype/Ssp/Year^d^	H2 (%)^e^
DTH (days)	2018	48.44	8.12	31–62	16.76	***/***/***	94.0
	2019	53.75	9.07	35–72	16.87		
	2021	51.88	8.86	33–71	17.08		
	2022	46.89	6.50	34–63	13.87		
PH (cm)	2018	105.0	22.53	60–150	21.46	***/***/***	94.3
	2019	135.6	27.04	70–190	19.94		
	2021	125.6	29.02	60–175	23.11		
	2022	119.5	24.74	56–171	20.70		
FLANG	2018	1.550	0.52	1–3	33.83	***/***/***	78.4
	2019	2.820	1.14	1–4	40.43		
	2021	2.188	0.83	0–3	38.08		
FLE	2018	1.401	0.53	1–4	37.82	***/***/***	69.2
	2019	2.712	0.80	1–4	29.50		
	2021	2.551	1.13	0–4	44.30		
FLL (cm)	2018	19.08	3.75	8.5–28.6	19.65	***/***/***	87.2
	2019	27.67	5.95	12.2–41.6	21.50		
	2021	25.74	4.70	13.8–38.8	18.26		
	2022	24.50	5.35	12.8–39.3	21.82		
FLW (cm)	2018	1.462	0.19	0.9–2.1	13.00	***/***/***	80.0
	2019	1.779	0.23	1.2–2.6	12.93		
	2021	1.607	0.24	0.9–2.3	14.93		
	2022	1.657	0.25	1–2.3	14.86		
FLA	2018	21.896	5.98	9.09–42.27	27.31	***/***/***	82.1
	2019	36.857	10.34	15.98–69.35	28.05		
	2021	31.760	8.57	12.94–56.1	26.98		
	2022	30.947	8.45	13.07–53.08	27.32		
SL (cm)	2018	8.60	4.42	3.6–19.9	51.40	***/***/***	98.1
	2019°	14.53	3.10	8.1–23	21.34		
	2021	10.12	4.71	3.6–20.3	46.54		
	2022	10.39	4.41	4.1–20.3	42.44		
GNS	2018	45.06	12.87	15–87.2	28.56	***/***/***	86.6
	2019°	43.76	10.37	23.5–72.6	23.70		
	2021	50.01	14.71	26–110.2	29.41		
	2022	52.31	17.75	20.2–112.6	33.93		
GWS (g)	2019°	1.760	0.42	0.72–3.01	23.86	***/***/***	79.3
	2021	1.972	0.56	0.94–3.82	28.40		
	2022	1.885	0.53	0.74–3.61	28.12		
SNS	2018	20.66	2.54	15.2–27.8	12.29	***/***/***	86.5
	2019°	23.72	2.95	17.8–30.8	12.44		
	2021	20.83	3.85	11.2–31.4	18.48		
	2022	22.65	3.44	14.5–32.5	15.19		
FSNS	2019°	21.62	3.05	16–29.4	14.11	***/***/***	87.9
	2021	19.87	3.59	11.2–30.4	18.07		
	2022	21.39	3.35	13.6–31.8	15.66		
SW (g)	2019°	2.669	0.63	1.08–4.44	23.60	***/***/**	76.1
	2021	2.753	0.69	1.31–4.95	25.06		
	2022	2.593	0.63	1.01–4.38	24.30		
SD^	2019°	17.33	5.43	11.3–34.2	31.33	***/***/***	98.7
	2021	25.04	10.78	10.19–52.91	43.05		
	2022	25.97	11.07	11.18–54.9	42.63		
TGW (g)	2021	39.72	6.84	24.25–58.6	17.22	ns/ns/***	61.0
	2022	35.43	6.07	21.50–52.50	17.12		
CHLs	2018	32.904	5.49	17.98–49.63	16.68	***/***/***	61.2
	2019	36.749	4.00	27.15–48.87	10.88		
	2021	43.501	3.98	32.92–56.20	9.15		
	2022	41.962	4.90	29.93–59.39	11.68		
CHLi	2018	32.757	5.46	18.69–48.95	16.67	***/***/***	62.0
	2019	36.504	4.10	26.75–50.21	11.23		
	2021	43.312	3.99	32.52–56.4	9.21		
	2022	41.935	4.91	29.25–59.89	11.71		
FLVs	2018	1.544	0.13	1.27–1.93	8.42	***/***/***	60.7
	2019	1.426	0.11	0.93–1.71	7.71		
	2021	1.349	0.15	0.89–1.68	11.12		
	2022	1.381	0.14	0.89–1.69	10.21		
FLVi	2018	1.559	0.13	1.23–1.94	8.34	***/***/***	59.0
	2019	1.533	0.11	1.22–1.90	7.18		
	2021	1.547	0.11	1.19–1.99	7.11		
	2022	1.514	0.12	1.12–1.80	7.64		
NBIs	2018	21.682	4.69	11.71–34.27	21.63	***/***/***	59.7
	2019	26.215	4.00	17.48–44.37	15.26		
	2021	33.085	4.93	20.68–48.05	14.90		
	2022	31.154	5.38	18.46–47.49	17.26		
NBIi	2018	21.380	4.72	11.89–37.19	22.08	***/***/***	57.6
	2019	24.125	3.56	15.71–36.64	14.76		
	2021	28.350	3.44	19.05–37.87	12.13		
	2022	28.070	4.17	16.44–40.05	14.86		

* DTH: days to heading; PH: plant height; FLANG: leaf angle; FLE: leaf elasticity; FLL: leaf length; FLW: leaf width; FLA: leaf area; SL: spike length; GNS: number of grains per spike; GWS: grain weight per spike; SNS: spikelet number per spike; FSNS: fertile spikelet number per spike; SW: spike weight; SD: spike density; SD^: derived spike density; TGW: by thousand grain weight; CHLs: chlorophyll content on superior leaf; CHLi: chlorophyll content on inferior leaf; FLVs: flavonoids content on superior leaf; FLVi: flavonoids content on inferior leaf; NBIs: CHLs/FLAs; NBIi: CHLi/FLVi.

**, *** Significance at *p* < 0.01, *p* < 0.001, respectively.

ns: not significant.

a Number of accessions/replicas tested: 151/2 in 2018, 190/3 in 2019, and 190/2 in 2021 and 2022.

b Standard deviation.

c Coefficient of variation.

d Only in ssp. *macha*, *spelta*, and *vavilovii*.

e Broad-sense heritability.

### 3.2 Genotyping for interesting loci

The allelic state at 16 known genes for plant height (*Rht8* and *Rht-B1a*), photoperiod responsiveness (*Ppd-D1a*), vernalization (*VRND4*), and genes for quality traits such as grain protein content (*Gcp-B1*), hardness (*Pinb-D1*), and grain weight (*GW2-6A*, *Sus1-7A*, *Sus1-7B*, *Sus2-2A*, *Sus2-2B*, *CKX6-D1*, *GS-D1-7D*, *Cwi-A1*, *Cwi-4A*, and *Cwi-5D*) was assessed (**Supplementary Table S3**). The genotyping data at these genes across the entire *Ta*-ssp panel are available in **Supplementary Table S6A**, while graphs reporting the allelic distribution in different ssp (except for the single *vavilovii* accession) and in the entire panel are presented in **Supplementary Table S6B**.

The great majority of the accessions carry the wild-type allele for *Rht8* and *Rht-B1a* (95.%2 and 93.1%), are photoperiod sensitive (92.9%), are soft type (92.6%), and do not have *Gpc-B1* (86.8%); this latter gene is carried only by 1/3 of the *spelta* accessions (with few other exceptions). *VRND4* was detected only in *sphaerococcum* at 92%, as already observed by Kippes et al. (2015) (**Table 3**). Eleven genes with effects on yield-related traits were tested, for some of which a significant correlation between a specific allelic variant (favorable allele) and an increase in TGW or grain number/size is known from the literature (**Supplementary Table S3**).

**Table 3:** Distribution of the alternative allelic variants at known genes among the accession intra-subspecies (ssp. *vavilovii* is not included) and in the *Ta*-ssp panel (expressed in %).

Gene	Allele	*Aestivum* (15)	*Compatum* (58)	*Sphaerococcum* (26)	*Spelta* (66)	*Macha* (24)	*Ta*-ssp panel (189)	% *Ta-*ssp panel
*Rht8*	dw	5	3	0	1	0	9	4.8
	wt	10	55	26	64	24	179	95.2
*Rht-B1a*	dw	8	3	0	2	0	13	6.9
	wt	7	55	26	64	24	176	93.1
*Ppd-D1*	Phot. Insens.	7	3	1	2	0	13	7.1
	Phot. Sens.	8	55	25	60	23	171	92.9
*VRND4*	VRND4	0	0	24	0	0	24	13.1
	No VRND4	14	59	2	60	24	159	86.9
*Pinb-D1*	Hard	4	3	1	1	5	14	7.4
	Soft	11	55	25	65	19	175	92.6
*Gpc-B1*	Gpc-B1	0	1	0	22	2	25	13.2
	No Gpc-B1	15	57	26	44	22	164	86.8
*GW2-6A*	Hap-A *	6	6	0	22	2	36	19.1
	Hap-G	8	52	26	44	22	152	80.9
*Sus1-7A_a*	Hap-1	5	43	4	25	8	85	45.5
	Hap-2	10	15	22	41	14	102	54.5
*Sus1-7A_b*	Hap-1	13	44	23	58	21	159	85.9
	Hap-2	1	14	3	6	2	26	14.1
*Sus1-7B*	Hap-C	3	16	15	63	2	99	40.4
	Hap-T	12	42	11	60	21	146	59.6
*Sus2-2A*	Hap-A *	10	48	1	19	22	100	57.1
	Hap-G	3	7	25	39	1	75	42.9
*Sus2-2B*	Hap-H *	5	18	21	2	1	47	26.7
	Hap-L	9	38	4	58	20	129	73.3
*CKX6-D1*	Hap-A *	2	1	0	0	0	3	1.6
	Hap-B	13	57	26	66	24	186	98.4
*GS-D1-7D*	GS-D1a *	10	19	23	54	20	126	68.1
	GS-D1b	5	38	3	9	4	59	31.9
*Cwi-A1*	Cwi-A1a *	10	29	5	54	11	109	57.7
	Cwi-A1b	5	29	21	12	13	80	42.3
*Cwi-4A*	Hap-C	13	43	26	65	6	153	81.0
	Hap-T *	2	15	0	1	18	36	19.0
*Cwi-5D*	Hap-C	15	50	17	60	24	166	88.8
	Hap-G *	0	8	9	4	0	21	11.2

* Favored allelic variant.

The majority of panel accessions carry the same allelic variant for many of these genes, and it is usually not the favorable one (highlighted with asterisk in **Table 3**) is the same for most of the panel, with only two exceptions: *Sus2-2A* and *GS-D1-7D*, for which the favorable alleles Hap-A (higher TGW) and GS-D1a (higher TGW and longer grain) are present in 57.1%, and 68.1% of the panel, respectively. The predominant allele, for each individual gene, turns out to be the same within the different subspecies as well, except for the predominance of Hap-1 for *Sus1-7A* in *compactum*, Hap-G for *Sus2-2A* in *sphaerococcum* and *spelta*, Hap-H for *Sus2-2B* in *sphaerococcum*, GS-D1b for *GS-D1-7D* in *compactum*, and Hap-T for *Cwi-4A* in *macha*.

### 3.3 SNP markers and physical coverage

Genotyping of the 190 wheat accessions with two Illumina arrays (20K and 25K) resulted in a raw dataset of 24,398 SNP markers that were filtered for MAF (≥5%) and missing data (≤10%). Moreover, those markers that failed in all genotypes were removed (461), resulting in a set of 20,729 useful SNPs. All genotypes were retained since none of them had more than 5% of the missing data.

For the analysis of genetic diversity, 6,088 other markers were excluded, owing to the absence of a hit or no unique map position according to the reference genome sequence of Chinese Spring RefSeq v2.1 (https://urgi.versailles.inra.fr/blast_iwgsc/). A total of 14,640 informative SNPs were retained (**Supplementary Table S7A**) distributed on the homoeologous genomes in this way: 41% on the A-genome, 46% on the B-genome, and 13% on the D-genome.

The total physical distance covered by these markers was 14,105.3 Mb (the real length of reference wheat genome is 14,462.2 Mb), with chromosome 6D having the lowest coverage (493.8 Mb on 503.6 Mb) and chromosome 3B having the highest (851.8 Mb on 866.1 Mb). Genomes A and B show 1 SNP per 0.83 Mb and 0.78 Mb, respectively, while the interval between two consecutive SNPs on genome D was higher: 1.99 Mb on average and up to 5.3 Mb on chromosome 4D. The highest number of markers was detected in chromosomes 5B, 3B, and 1B (1,158, 1,127, and 1,082 SNPs, respectively), whereas chromosomes 4D and 3D showed the lowest number of located loci (97 and 212, respectively; **Supplementary Table S7B**).

### 3.4 Population structure and linkage disequilibrium

**Figure 2:**
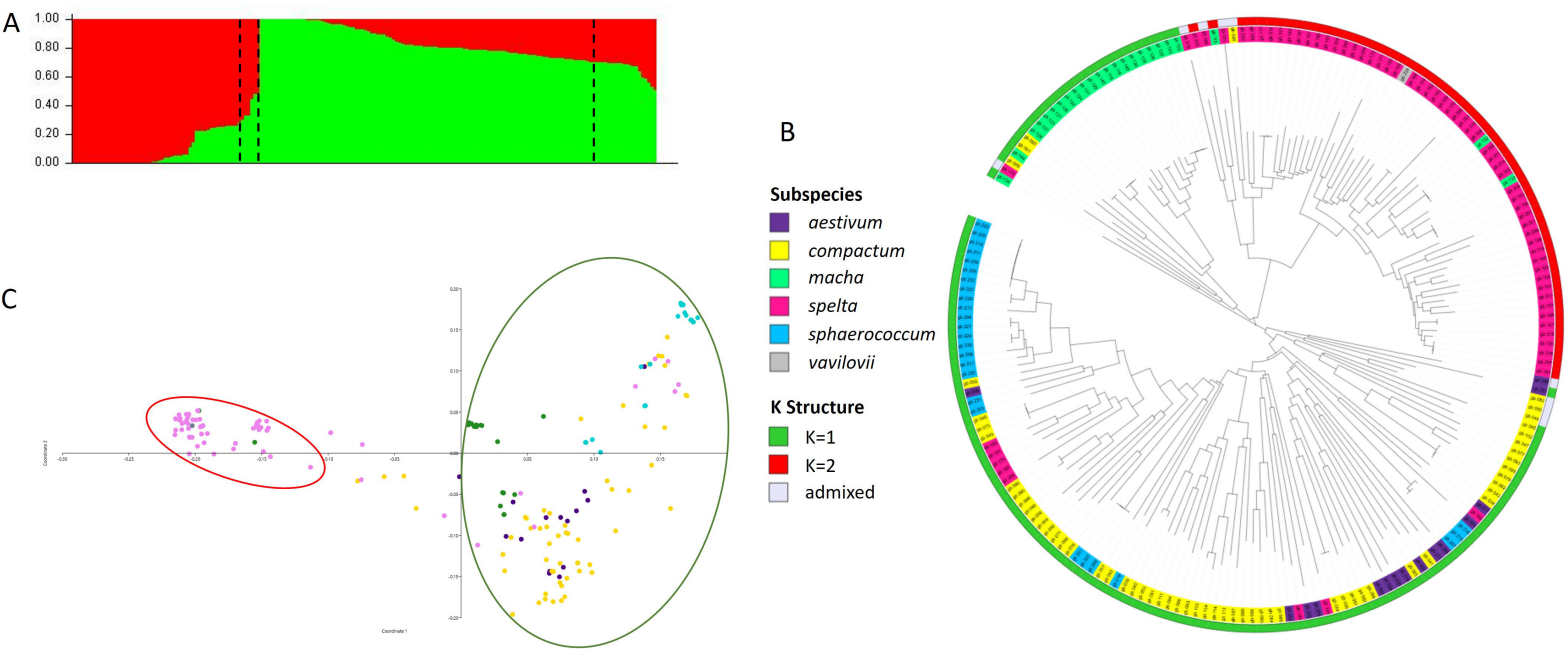
Analysis of population structure of the *Ta*-ssp panel. **(A)** Population structure based on STRUCTURE when *K* = 2. **(B)** Neighbor-joining tree and **(C)** PCoA (the clustering in the legend is based on the subspecies and the STRUCTURE analysis).

Several methods were used to investigate the population structure of the *Ta*-ssp panel. The results of the N-J, PCoA, and structure analyses are shown in **Figure 2**. The most likely structure model assigned 181 accessions (corresponding to 95.3% of the panel) to two clusters (**Supplementary Figure S2**): K2, containing most of the *spelta* accessions (81.8%) plus the single *vavilovii* (red cluster), and K1, which included the other four subspecies (green cluster) (**Figure 2A** and **Supplementary Table S1**). A small percentage (4.7%) of accessions were admixed: four *spelta*, four *compactum*, and one *aestivum*.

**Figure 3:**
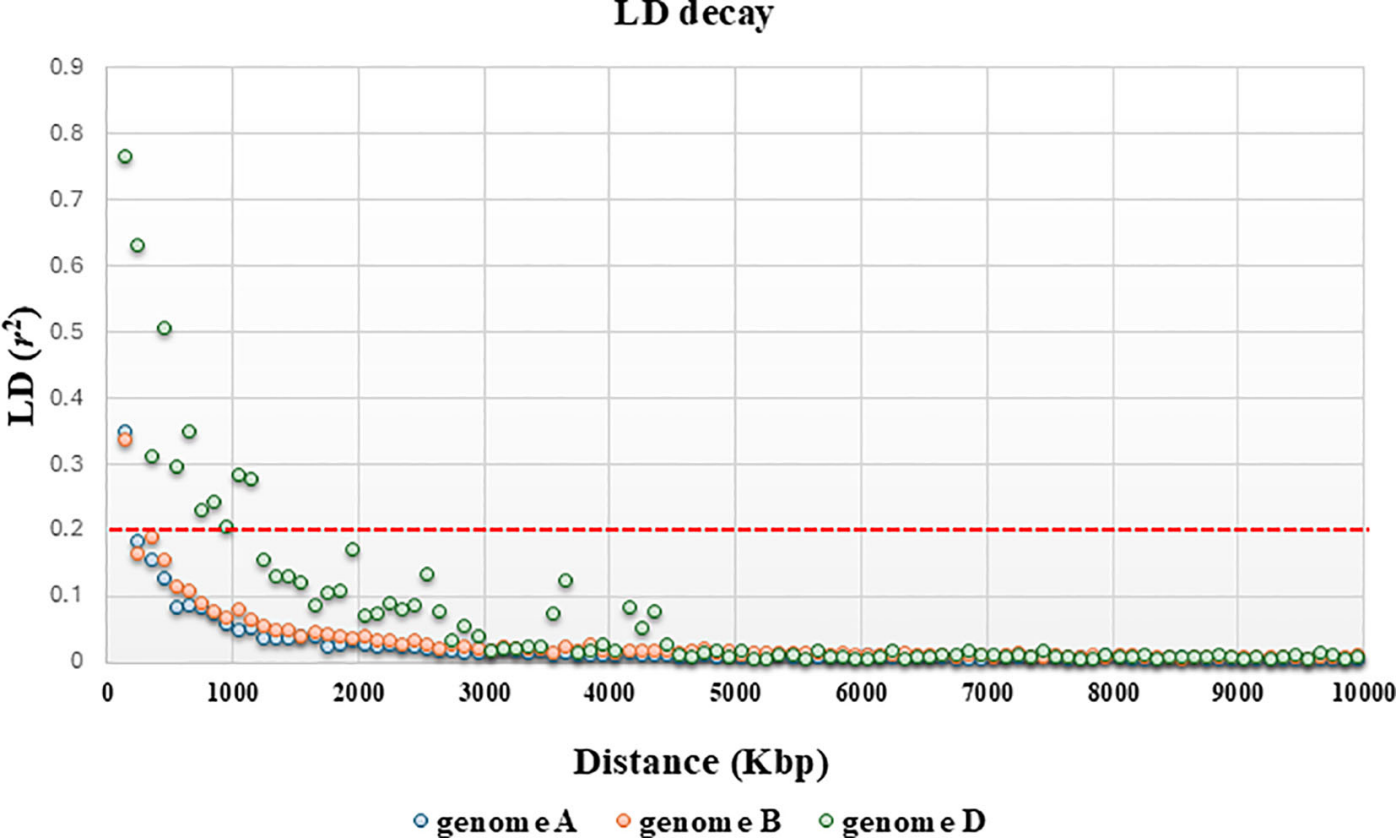
Genome-wide LD decay across A, B, and D subgenomes of hexaploid wheat: a focus along a distance of 10,000 bp. The *Y*-axis represents squared correlation coefficients (*r* ^2^) and the values of *X*-axis depict genetic distance in Kbp. The horizontal red line shows the threshold *r* ^2^ value for unlinked markers.

The clustering derived from the N-J tree (**Figure 2B**) is only partly overlapped to structure analysis. Three main roots were highlighted, one of which corresponds entirely to K2 and all *spelta* (except for eight accessions, the only ones of Asian origin—Afghanistan, Iran, and Tajikistan), while K1 is divided into two roots, one of which includes 87.5% of the *macha* accessions, plus three *compactum*, while the other one contains all *sphaerococcum* and most of the *compactum* along with the few remaining *aestivum*. As for PCoA, the first two principal coordinates accounted for 22.88% and 10.83% of the molecular variance of the panel, respectively, showing clustering consistent with that suggested by structure (**Figure 2C**). The extent of LD was calculated using 3,362 SNPs for subgenome A, 3,787 SNP for subgenome B, and 1,316 SNPs for subgenome D. Based on a critical threshold of *r* ^2^ value of 0.2, the LD decay distance on all chromosomes in the *Ta*-ssp panel was approximately 378.6 Kb, which is the average value over the 21 chromosomes (**Table 4**, **Figure 3**, and **Supplementary Figure S3**). A heterogeneous distribution was observed among the different genomes: the highest LD decay distance of 750 Kb was found in subgenome D, while subgenomes A and D have a lower and similar LD decay distance of 178.6 and 207.1 Kb, respectively.

**Table 4:** Analysis of chromosome-wise and genome-wise LD decay.

Chromosome subgenome	Critical decay distance° (Kbp)	Chromosome subgenome	Critical decay distance° (Kbp)	Chromosome subgenome	Critical decay distance° (Kbp)
1A	150	1B	150	1D	1,150
2A	150	2B	250	2D	850
3A	150	3B	250	3D	750
4A	150	4B	350	4D	750
5A	250	5B	150	5D	950
6A	150	6B	150	6D	450
7A	250	7B	150	7D	350
A	178.6	B	207.1	D	750
ABD 378.6					

°Corresponding to *r* ^2^ values of 0.2.

### 3.5 Association analysis

The output of GWAS was depicted in Manhattan plots for each trait in **Supplementary Figure S4** and summarized in **Supplementary Table S8**, in which all MTAs found to be linked to a given trait with a −log_10_ (*p*-value) ≥ 3.5 were listed with those SNPs together with colocalization with MTAs, QTL (quantitative trait locus), or genes known in literature to be linked with the same traits. MTA regions were identified according to the LD decay calculated for each chromosome and are listed in **Table 5**, defined as trait acronym.chr number/region number. The number of MTAs included in the region was reported, besides the peak marker.

**Table 5:** Summary of the marker–trait association regions.

	Physical position (bp)^c^				Peak marker		
MTA region^a^	SNP^b^	Start	End	Interval size	SNP	Position (Kb)	−log_10_(*p*-value)
DTH.3B/1	1	–	–	–	AX-158538277	229, 507, 213	3.893
DTH.5A/1	4	582, 691, 526	583, 355, 014	663, 488	AX-89311025	582, 824, 068	4.493
DTH.6B/1	1	–	–	–	AX-94900754	163, 837, 659	3.729
DTH.6B/2	2	479, 321, 198	479, 333, 211	12, 013	RFL_Contig3644_757	479, 333, 211	4.121
PH.1A/1	1	–	–	–	AX-94895904	305, 566, 968	3.501
PH.1A/2	1	–	–	–	wsnp_Ex_c14866_22995097	344, 966, 281	4.285
PH.4B/1	1	–	–	–	AX-158583339	15, 020, 330	3.633
PH.5B/1	1	–	–	–	wsnp_Ra_c2105_4092507	558, 279, 025	3.760
PH.6D/1	1	–	–	–	RAC875_c18002_58	153, 174, 444	4.211
FLANG.2A/1	1	–	–	–	AX-158537909	711, 940, 547	3.810
FLANG.2A/2	4	732, 751, 146	733, 288, 303	537, 157	BS00057060_51	733, 278, 001	4.804
FLANG.2B/1	1	–	–	–	BS00067907_51	730, 761, 972	4.316
FLANG.2D/1	2	13, 640, 846	13, 653, 433	12, 587	BS00063632_51	13, 640, 846	3.841
FLANG.3A/1	1	–	–	–	AX-158523900	72, 682, 937	4.098
FLANG.5A/1	1	–	–	–	wsnp_Ex_c44164_50292954	595, 164, 329	3.909
FLANG.5A/2	1	–	–	–	Tdurum_contig13810_485	649, 072, 000	3.977
FLANG.5D/1	1	–	–	–	wsnp_RFL_Contig2265_1693968	476, 627, 848	3.909
FLANG.7B/1	3	721, 873, 731	722, 508, 527	634, 796	AX-158567624	721, 873, 731	5.076
FLE.6B/1	1	–	–	–	BS00092845_51	13, 786, 271	3.931
FLL.3B/1	1	–	–	–	AX-158548385	571, 508, 039	3.682
FLL.4B/1	1	–	–	–	AX-158618889	2, 301, 121	4.088
FLL.4B/2	1	–	–	–	IAAV1120	5, 044, 665	3.511
FLL.6B/1	1	–	–	–	AX-89493979	479, 321, 198	4.271
FLW.1B/1	1	–	–	–	BS00083533_51	75, 661, 021	3.538
FLW.1B/2	1	–	–	–	JD_c954_2440	648, 250, 369	3.837
FLW.5B/1	1	–	–	–	Excalibur_c23452_401	693, 842, 938	3.780
FLW.7D/1	1	–	–	–	IACX7421	630, 908, 232	3.713
FLA.1B/1	1	–	–	–	JD_c954_2440	648, 250, 369	4.190
FLA.6B/1	1	–	–	–	AX-89493979	479, 321, 198	3.550
FLA.7B/1	1	–	–	–	RAC875_c30453_292	556, 791, 009	3.909
AP.1B/1	1	–	–	–	BS00052089_51	391, 192, 268	3.754
AP.3B/1	1	–	–	–	AX-110941435	778, 713, 102	4.031
AP.4A/1	2	604, 570, 787	604, 570, 789	2	TGWA25K-TG0192	604, 570, 787	3.819
AP.4B/1	1	–	–	–	AX-158550208	661, 745, 396	3.609
**AP.4D/1**	1	–	–	–	**RAC875_c8642_231**	**509, 763, 403**	**6.282**
AP.5A/1	2	700, 302, 152	700, 306, 955	4, 803	tplb0049a09_1302	700, 306, 955	5.611
AP.7A/1	1	–	–	–	AX-158559861	83, 956, 363	3.806
AP.7A/2	1	–	–	–	Ku_c43151_811	723, 992, 356	3.873
AP.7B/1	2	669, 225, 649	669, 248, 013	22, 364	Tdurum_contig44993_359	669, 225, 649	3.853
GLC.1A/1	5	1, 206, 149	1, 341, 356	135, 207	wsnp_Ra_c11211_18227610	1, 341, 356	5.158
**GLC.1B/1**	2	4, 532, 360	4, 533, 010	650	**AX-158540295**	**4, 532, 360**	**10.086**
GLC.1D/1	1	–	–	–	Kukri_c37738_417	3, 183, 262	4.671
GLC.3B/1	2	745, 956, 445	745, 957, 777	1, 332	Excalibur_c2850_126	745, 956, 445	4.689
GC.1B/1	4	632, 633, 016	633, 144, 183	511, 167	Tdurum_contig41945_748	633, 129, 623	4.728
GC.2A/1	1	–	–	–	AX-94541185	452, 564, 062	3.680
GC.2A/2	1	–	–	–	AX-94661746	722, 711, 222	3.630
GC.2A/3	1	–	–	–	AX-94611746	753, 940, 370	4.619
**GC.2B/1**	1	–	–	–	**RAC875_c18928_455**	**791, 641, 125**	**7.406**
GC.3A/1	1	–	–	–	AX-95232910	650, 245, 411	3.637
GC.6A/1	2	599, 246, 668	599, 246, 811	143	wsnp_Ex_c20457_29526260	599, 246, 811	5.033
GC.6B/1	1	–	–	–	BS00028997_51	652, 618, 443	4.128
GC.6D/1	1	–	–	–	BS00066209_51	315, 767, 532	4.037
GC.7B/1	1	–	–	–	wsnp_Ex_c24376_33619527	36, 162, 232	3.635
GC.7D/1	1	–	–	–	BS00079734_51	633, 897, 302	3.754
**SS.2A/1**	1	–	–	–	**AX-94541185**	**452, 564, 062**	**6.508**
SS.2D/1	1	–	–	–	AX-158562171	187, 629, 499	4.680
**SS.2D/2**	1	–	–	–	**AX-158522249**	**360, 172, 914**	**5.790**
SS.4B/1	1	–	–	–	AX-158618805	55, 561, 370	3.626
SS.5A/1	1	–	–	–	AX-111607045	701, 983, 810	3.540
SS.6B/1	1	–	–	–	AX-158559508	12, 388, 259	3.709
SS.6B/2	1	–	–	–	AX-89493979	479, 321, 198	3.809
SS.7A/1	1	–	–	–	IAAV6170	542, 778, 435	3.663
**SL.2A/1**	1	–	–	–	**AX-94541185**	**452, 564, 062**	**6.274**
SL.2D/1	1	–	–	–	AX-158562171	187, 629, 499	3.843
SL.2D/2	1	–	–	–	AX-89640158	342, 959, 863	3.768
**SL.2D/3**	1	–	–	–	**AX-158522249**	**360, 172, 914**	**6.567**
SL.2D/4	2	371, 893, 971	372, 173, 004	279, 033	AX-108781921	371, 893, 971	3.707
SL.5B/1	1	–	–	–	AX-158533884	547, 867, 553	3.516
SNS.3B/1	1	–	–	–	Kukri_c3168_521	159, 428, 491	4.067
SNS.5B/1	1	–	–	–	BS00024993_51	7, 987, 279	3.770
SNS.7B/1	1	–	–	–	AX-109821362	9, 194, 827	3.563
SNS.7B/2	1	–	–	–	RAC875_c21489_908	640, 204, 671	4.229
SNS.7D/1	1	–	–	–	IACX7421	630, 908, 232	3.518
FSNS.3B/1	1	–	–	–	Kukri_c3168_521	159, 428, 491	3.804
FSNS.6B/1	1	–	–	–	Excalibur_rep_c103447_433	220, 151, 275	4.024
**SD.2A/1**	1	–	–	–	**AX-94541185**	**452, 564, 062**	**7.389**
SD.2D/1	1	–	–	–	AX-89640158	342, 959, 863	3.637
**SD.2D/2**	1	–	–	–	**AX-158522249**	**360, 172, 914**	**7.569**
SD.2D/3	2	371, 893, 971	372, 173, 004	279, 033	AX-108781921	371, 893, 971	3.506
SD.3A/1	1	–	–	–	AX-109905851	82, 098, 377	4.163
SD.4A/1	1	–	–	–	AX-109855651	593, 352, 866	3.601
SD.5B/1	1	–	–	–	AX-110576712	708, 947, 944	3.532
SD.6B/1	2	486, 002, 460	486, 002, 659	199	wsnp_CAP11_c3599_1741800	486, 002, 460	4.014
**SD^.2A/1**	1	–	–	–	**AX-94541185**	**452, 564, 062**	**7.502**
SD^.2D/1	1	–	–	–	AX-89640158	342, 959, 863	3.667
**SD^.2D/2**	1	–	–	–	**AX-158522249**	**360, 172, 914**	**7.668**
SD^.2D/3	2	371, 893, 971	372, 173, 004	279, 033	AX-108781921	371, 893, 971	3.726
SD^.6B/1	1	–	–	–	AX-158588957	447, 414, 300	3.642
SD^.6B/2	1	–	–	–	AX-158566374	479, 460, 461	3.533
GNS.3A/1	1	–	–	–	IACX5844	103, 079, 471	3.725
GNS.3B/1	1	–	–	–	IACX5776	6, 077, 630	3.787
GNS.3B/2	1	–	–	–	BS00011570_51	79, 878, 365	4.169
GNS.3B/3	1	–	–	–	wsnp_Ex_c5657_9942445	149, 396, 871	4.351
GNS.6A/1	1	–	–	–	BS00043716_51	233, 324, 558	3.783
GNS.6B/1	2	152, 098, 069	152, 098, 568	499	RAC875_c54818_481	152, 098, 069	3.859
GNS.7B/1	1	–	–	–	AX-94385458	674, 224, 844	3.616
GWS.3A/1	1	–	–	–	tplb0053a24_1247	8, 670, 672	3.594
GWS.5B/1	1	–	–	–	AX-158565542	464, 082, 908	3.740
GWS.7D/1	1	–	–	–	BS00021745_51	633, 897, 316	3.745
TGW.5B/1	1	–	–	–	Kukri_c31961_630	284, 765, 351	3.836
TGW.6A/1	1	–	–	–	BS00021704_51	614, 864, 750	4.220
CHLs.6A/1	1	–	–	–	tplb0056d17_78	557, 071, 115	3.653
CHLs.6A/2	1	–	–	–	CAP12_c118_95	557, 483, 060	4.572
CHLs.6D/1	1	–	–	–	RAC875_c25839_225	313, 754, 095	4.744
CHLi.6A/1	1	–	–	–	tplb0056d17_78	557, 071, 115	3.623
CHLi.6A/2	1	–	–	–	CAP12_c118_95	557, 483, 060	4.622
CHLi.6D/1	1	–	–	–	RAC875_c25839_225	313, 754, 095	4.938
FLVs.4A/1	1	–	–	–	AX-158524633	538, 764, 962	4.100
FLVs.4A/2	1	–	–	–	AX-109937816	567, 176, 093	3.926
FLVi.2D/1	1	–	–	–	AX-95240055	580, 289, 948	3.598
FLVi.4A/1	1	–	–	–	IAAV4351	593, 036, 071	3.538
FLVi.4B/1	1	–	–	–	AX-158583339	15, 020, 330	4.131
FLVi.4B/2	1	–	–	–	AX-89747834	26, 180, 807	3.532
FLVi.5A/1	2	479, 293, 861	479, 318, 299	24, 438	BS00107192_51	479, 293, 861	3.582
FLVi.7A/1	2	6, 862, 753	6, 863, 608	855	Kukri_c99396_111	6, 862, 753	3.905
FLVi.7B/1	1	–	–	–	AX-158543936	666, 545, 771	3.573
FLVi.7D/1	1	–	–	–	AX-110425310	13, 402, 322	3.509
NBIs.2D/1	1	–	–	–	AX-111574425	34, 426, 508	3.501
NBIs.5A/1	1	–	–	–	AX-94723827	678, 693, 050	4.211
NBIs.5B/1	1	–	–	–	AX-158565538	592, 792, 435	3.958
NBIs.5B/2	1	–	–	–	AX-158525184	714, 623, 007	3.508
NBIs.6A/1	1	–	–	–	CAP12_c118_95	557, 483, 060	3.765
NBIi.2B/1	1	–	–	–	AX-94392210	658, 334, 699	3.909
NBIi.4B/1	1	–	–	–	AX-158583339	15, 020, 330	4.199
NBIi.5A/1	1	–	–	–	AX-94723827	678, 693, 050	3.986
NBIi.6A/1	1	–	–	–	CAP12_c118_95	557, 483, 060	4.087
NBIi.6D/1	1	–	–	–	RAC875_c25839_225	313, 754, 095	4.440
NBIi.7D/1	1	–	–	–	BS00022203_51	60, 593, 072	3.609

a Trait.Chromosome/number of MTA (marker–trait association) region on that chromosome. The MTA exceeding Bonferroni’s threshold and with an FDR (false discovery rate) less than 0.05 (Benjamini and Hochberg, 1995) is denoted in bold.

b Number of SNPs in the MTA region.

c Physical position and interval size of the MTA region in base pairs on IWGSC Ref Seq 2.1.

The GWAS on DTH resulted in four associated regions on three different chromosomes (3B, 5A, and 6B) with a −log_10_ (*p*-value) ranging from 3.729 to 4.493.

The GWAS detected 58 associated regions for morphology-related traits (AP, PH, FLANG, FLE, FLL, FLW, FLA GLC, GC, and SS): five MTAs resulted in being highly significant over Bonferroni threshold (in bold in **Table 5**).

A total of 39 MTA regions were detected for yield-related traits. Spike length and spike density (calculated by both methods, see Material and Methods) shared four MTA regions, two of them on chromosomes 2A and 2D identified by strongly associated peak markers (over Bonferroni significance): AX-94541185 and AX-158522249, respectively. The MTA associated with FSNS (at 159.4 Mb on chromosome 3B) was also detected for FSNS. Among the regions identified for spike density, measured by two different methods, there was a match between four MTA regions, two of which had a highly significant −log_10_ (*p*-value), greater than 7: that on chromosome 2A (at 452.6 Mb; AX-94541185) and that on chromosome 2D at 360.2 Mb (AX-158522249).

For physiological traits, the analysis yielded a total of 25 associated regions. CHLs and CHLi shared the same two MTA regions on chromosomes 6A and 6D, at 557.5 Mb and 313.8 Mb, respectively. Peak markers of MTA regions associated with multiple traits are summarized in **Supplementary Table S9**, and high-confidence (HC) genes located within the chromosome-specific LD (upstream and downstream) were identified and reported alongside their functional annotation on IWGSC RefSeq v2.1.

## 4 Discussion

### 4.1 Population structure of the subspecies collection

The hexaploid subspecies of bread wheat, apart from ssp. *aestivum*, have not undergone intensive selection, having been grown in isolated and not particularly attractive environments, so they can still exhibit a broad range of variation for many traits. There is a wealth of studies on GWAS in wheat, as summarized in Saini et al. (2022), but so far none has included such a significant number of accessions belonging to different hexaploid subspecies. Only Abrouk et al. (2021) investigated by association mapping a germplasm of 102 Central European spelt accessions, but only for the glume color trait. The panel set up in this study represents the first comprehensive resource of different hexaploidy subspecies, and it represents a valuable reservoir of new alleles and genes that are largely unexploited and potentially useful for wheat improvement.

The genotyping of our *Ta*-ssp panel resulted in the identification of 14,640 SNPs with a physical position on the Chinese Spring RefSeq v2.1 Reference genomic sequence. The distribution of these markers on the genome and among the subgenomes was consistent with previous studies (Aleksandrov et al., 2021; Balfourier et al., 2019; Leonova et al., 2020; Pang et al., 2020), as well as the lower coverage of subgenome D that is likely due to the latter’s low representation in the 25K SNP array (Rimbert et al., 2018) as a consequence of its known low polymorphism (Pont et al., 2019).

Results from the three clustering methods (N-J tree, PCoA, and structure analysis) showed the presence of two subpopulations: K2 comprising the *spelta* and K1 comprising the other remaining subspecies. Notably, *spelta* of Asian origin (a total of five accessions, from Afghanistan, Tajikistan, and Iran) were separated from all the others as previously observed by Abrouk et al. (2021) and Hyun et al. (2020) and were clustered in K1 among ssp. *compactum*. These strong differences between Asian and European spelt wheats had already been pointed out (Abrouk et al., 2021). In addition, the two roots in K2 divide the group spelt from the Iberian Peninsula (eight accessions) from those of Central Europe, again in agreement with Abrouk’s study (2021). Ssp. *macha*, the other spelt form, although found in K1, is grouped in a separate root of the N-J tree and is closer to European *spelta* than to other subspecies. Integration of genotyping data from the present study with those from emmer wheat and common wheat collections representative of genetic diversity could direct future sequencing projects aimed at unraveling the evolutionary dynamics existing among different hexaploid subspecies and with tetraploid genome donors. The few *aestivum* accessions considered in this study, which were not selected for this purpose, do not allow us to elucidate these relationships at present.

### 4.2 Significant associations in the GWAS and comparison with previous studies

The GWAS analysis, performed with correction for the population structure, includes all polymorphic markers (20,729) and not only those physically mapped (14,640) through a very stringent BLAST analysis.

The pairwise LD (*r* ^2^) calculated for the *Ta*-ssp panel decayed to a value of less than 0.2 (generally assumed as an *r* ^2^ value between unlinked loci) at 378.6 Kb (as the average value across all chromosomes and genomes). This very short decay is somewhat expected in such a diverse panel, composed of landraces and wilds and not or only partially subjected to breeding; it is closer to that observed in einkorn (176 Kb, Volante et al., 2021) and in wild emmer (126 Kb, Mastrangelo et al., 2023) than to that in cultivated/modern common wheat (6.6 Mb in Hu et al., 2021; 5.8 Mb in Mazumder et al., 2024).

Since different subspecies were analyzed together, considerable phenotypic variability was observed in the *Ta*-ssp panel, especially for DTH, PH, FLA, SL, GS, and SD^. Significant effects were found for the year factor, that is why we decided to perform a GWAS analysis with the across-year BLUPs and maintain a threshold of −log_10_ (*p*-value) at medium stringency (≥3.5) for defining the associated MTAs. The marker set employed in this study allowed the identification of many loci associated with the measured traits. MTA regions were identified based on the LD decay of each individual chromosome. The rapid decay led, in some cases, to the definitions of MTAs that were quite close to each other: to exclude the idea that the linkage between these regions had been underestimated by using the chromosome mean LD value, LD between markers of the next MTA region was inspected.

These associated regions were inspected for colocalization with MTAs/QTL/genes with a known function previously identified in common wheat. The literature survey resulted in several overlaps reported in **Supplementary Table S8**.

The DTH.5A/1 locus (582.7 Mb to 583.4 Mb) identified by the peak marker AX-89311025 was located near the vernalization gene *Vrn-A1* (589.3 Mb).

The AP.5A/1 region on chromosome 5A (700.3–700.3 Mb) has been mapped in close proximity to the *B1* locus (698.7 Mb; Li et al., 2023), one of the dominant inhibitory genes for awn development in wheat, while the AP.5 B/1 on chromosome 5B (598.5-598.6 Mb; tplb0049a09_1302) is not far from AX-95632082, a marker previously linked to the presence of awn (607.7 Mb, Sheoran et al., 2019).

In detail, the peak marker of GLC.1B/1, AX-158540295 (4.5 Mb), which is highly correlated [−log_10_ (*p*-value) ≥ 10] with glume color, maps at the same location as AX-94980178 already associated with the same trait on chromosome 1B (Sheoran et al., 2019). The SL.5B/1 region defined by the peak marker AX-158533884 at 547.9 Mb is next to BS00087043_51 at 553.5 Mb already associated with spike length by Anuarbek et al. (2020).

The MTA SNS.7B/2 at 640.2 Mb (RAC875_c21489_908) is not far from an MTA previously identified for the same trait at 645.0 Mb (Muqaddasi et al., 2019). The Excalibur_c23452_401 marker on chromosome 5B at 693.8 Mb found to be associated with FLW is very close to an MQTL identified at 670.5–690.2 Mb by Kong et al. (2023) for the same leaf trait.

The colocalization between some of the MTAs observed in this study and some reported in previous GWAS supports the reliability of our analysis; on the other hand, the fact that, for most MTAs, there is no support from literature data suggests that the panel employed carries loci that are novel and therefore of potential interest.

### 4.3 Genomic regions affecting multiple traits

Specific attention was dedicated to MTA regions shared by several traits (**Supplementary Tables S8**, **S9**). Along chromosome 2D, there are four distinct MTA regions associated with three components of spike morphology that are strongly related to grain yield in wheat: spike shape, density, and length (Upadhyay, 2020; Liu et al., 2022; Halder et al., 2023). At 187.7 Mb, the SNP AX-158562171 is associated with SS and SL, while AX-89640158 (343 Mb), AX-158522249 (360.2 Mb), and AX-108781921 (371.9 Mb) are associated with SL, SD, and SD^. These three latter regions are of great interest, especially the MTA defined by AX-158522249, which resulted in being highly significant (exceeding the Bonferroni threshold) for all traits, including also SS (directly related with spike density). This MTA region corresponds to the genetic location of gene *C*, the locus responsible for spike compactness with strong effects on other key targets, such as the shape and size of spikes and the number of grains per spike. The *C* gene has been initially reported to map to the centromeric interval, and this has greatly hindered its physical mapping, discouraging potential gene cloning of it. By QTL mapping, Wen et al. (2022) mapped this gene more distal from the centromeric region but still in a quite wide interval along the 2D chromosome (370.12–406.29 Mb). Our GWAS approach, thanks to the small LD of the *Ta*-ssp panel characterized by multiple recombination events among numerous compact and non-compact accessions, resulted in a better definition of the interval around the *C* gene (1.7 Mb; 359.3 Mb–361 Mb). This region is very promising as a starting point for the isolation of the *C* gene through a map-based cloning method.

Another MTA (AX-94541185) shared between the same traits (SS, SL, SD, and SD^) with a very high significance was detected on chromosome 2A with no correspondence with any other region already detected in literature, as far as we know. In 2022, Yu et al. performed a genetic dissection of major QTL, in a bi-parental population, for spike compactness and length on homology group 2, but none of them overlap or are in the proximity of the novel regions that we identified here.

Other interesting MTA regions are on chromosome 1B at 648.2 Mb (JD_c954_2440 640) associated with FLL and FLA; on chromosome 6B at 479.3 Mb (AX-89493979) associated with FLL, FLA, and SS; and on chromosome 7D at 631 Mb (IACX7421) associated with both spikelet number per spike and spike flag leaf width. Finally, the two MTAs identified by CHLs, CHLi, and NBI on chromosomes 6A and 6D (at 557 and 313.8 Mb, respectively) are also noteworthy.

Generally, the traits assigned to the same genomic regions are supported by significant Pearson correlation values (**Supplementary Table S5**: significant positive or negative correlations were highlighted in blue and red, respectively), suggesting their regulation by multiple co-effect loci.

These regions could be the results of pleiotropic effects or robust physical linkage between underlying genes and deserve further investigations, a deeper genetic dissection, and candidate gene investigation.

Among the peak markers of the identified regions, some were fixed only in one subspecies, usually *macha* or *sphaerococcum*. For instance, all *sphaerococcum* carry the unfavorable allelic variant at GNS.3B/2 associated with fewer seeds per spike. Given the subspecies characteristics, these apparently obvious remarks support the reliability of our analyses.

Noteworthy is the GNS.6A/1 locus that identifies a novel MTA related to the number of seeds per spike, found in both 2021 and 2022. The peak marker of this MTA, BS00043716_51, segregates exclusively in *compactum* accessions. The number of grains per spike is significantly higher in those *compactum* accessions bringing the allelic variant “C” (89.8 and 78.8 in 2021 and 2022, respectively) compared to those with “T” (62.4 and 58.8), and to all the other subspecies (44.3 and 44.8, in 2021 and 2022, respectively) with the unfavorable allele “T”. The *compactum* accessions carrying this favorable allele “C” for GNS also showed higher grain weight per spike (GWS) (2.8 g, 2021–2022 mean value), compared to those with “T” (2.2 g) and to all the other subspecies (1.7 g). GNS correlated positively and significantly with GWS (0.83) in this study, but we did not find any significant MTA for GWS on chromosome 6A.

We identified two potential candidate genes underlying this GNS.6A/1 locus. One is *TaGW2-6A*, a gene encoding a ubiquitin ligase that negatively controls controlling grain size and weight (Geng et al., 2017) located on chromosome 6A at 240 Mb, not far from the peak marker of GNS.6A/1 (BS00043716_51) that maps at 233 Mb. The second potential candidate is the gene *TraesCS6A03G0476500*, which includes BS00043716_51 (Raw ID: *TraesCS6A02G188700*, chr6A: 233 324 244–233 328 477). This gene encodes for a monosaccharide-sensing protein 2 known to act in rice as a vacuolar transporter of glucose (Cho et al., 2010) and with a pivotal role in plant growth and development. According to the Wheat eFP Browser, the expression pattern of *TraesCS6A03G0476500* localizes in the shoot meristem, spike, and seed, with highest values in the endosperm. These observations point out the potential role of this gene in the determination of grain number per spike.

Taken together, these data, despite needing further investigation, confirm the potentiality hidden in the wheat subspecies for key traits related to yield potential.

## 5 Conclusion

The genetic and phenotypic characterization carried out in this work revealed the genetic basis underlying the hidden diversity for many agronomic traits present in wheat subspecies. A combination of favorable alleles for traits such as SL, GNS, SD, GWS, and TGW could assist the selection of accessions for pre-breeding purposes, as well as an effective deployment of new valuable genes or alleles into modern breeding varieties.

In addition, optimization of leaf shape traits, together with its spatial arrangement, and a high chlorophyll content could reduce individual response to shading and improve photosynthetic efficiency, thereby increasing yield. Further characterization of this genetic resource for resistance to biotic and abiotic stress is underway, in natural and controlled conditions, as a strategic step for efficient exploitation of this material in pre-breeding programs. Novel candidate genomic loci underlying several agronomic traits have been identified in hexaploid subspecies that, once validated, could leave alternative clues for the wheat research and breeding community. Genomic regions controlling multiple traits have also been identified that could be exploited for pyramiding favorable alleles for key traits related to yield.

## Data Availability

The datasets related on the phenotypic data of four field trials, BLUP values and the hapmap used for GWAS presented in this study available at figshare: https://doi.org/10.6084/m9.figshare.27931290.
