# Supplementary material for: Ancient diversity of Triticum aestivum subspecies as source of novel loci for bread wheat improvement
Source: Front Plant Sci. 2025 Apr 9;16:1536991. doi: 10.3389/fpls.2025.1536991 (PMC12014548; doi:10.3389/fpls.2025.1536991)

Supplementary Figure S1: Distribution of the traits intra ssp: barplots and boxplots for the four years.

TAA: ssp *aestivum*

TAC: ssp *compactum*

TAM: ssp *macha*

TASPE: ssp *spelta*

TASPH: ssp *sphaerococcum*

TAV: ssp *vavilovii*

Phenotyping Fiorenzuola 2018 (151 genotypes, 2 rep)

Data-points
DTH: 304
PH: 304
SL: 293

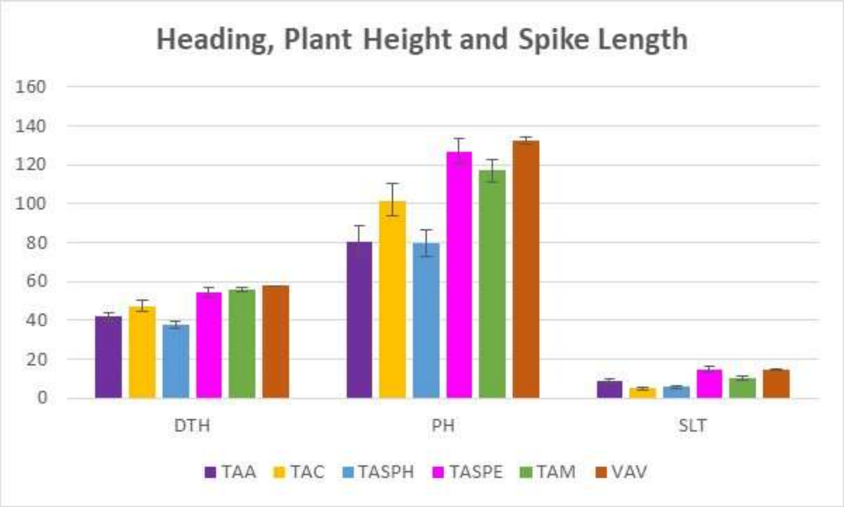

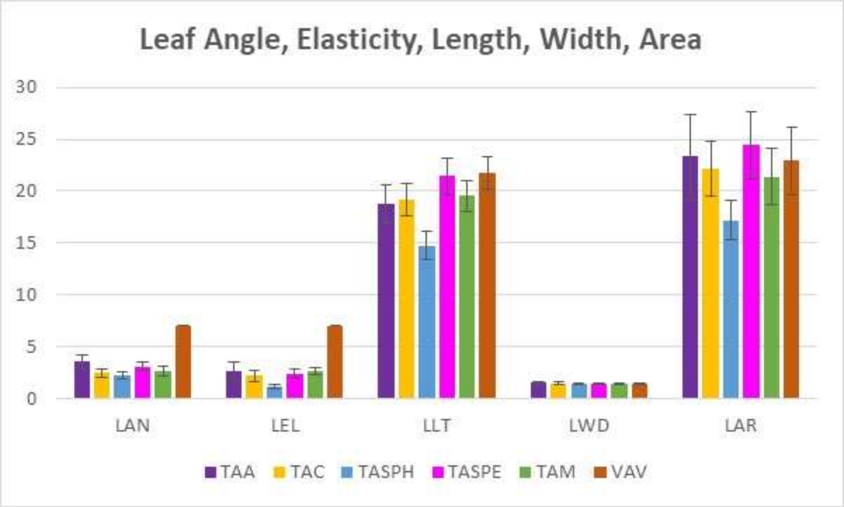

Data-points
FLANG: 304
FLE: 304
FLL: 271
FLW: 271
FLA: 271

Data-points
CHL: 292
FLV: 292
NBI: 292

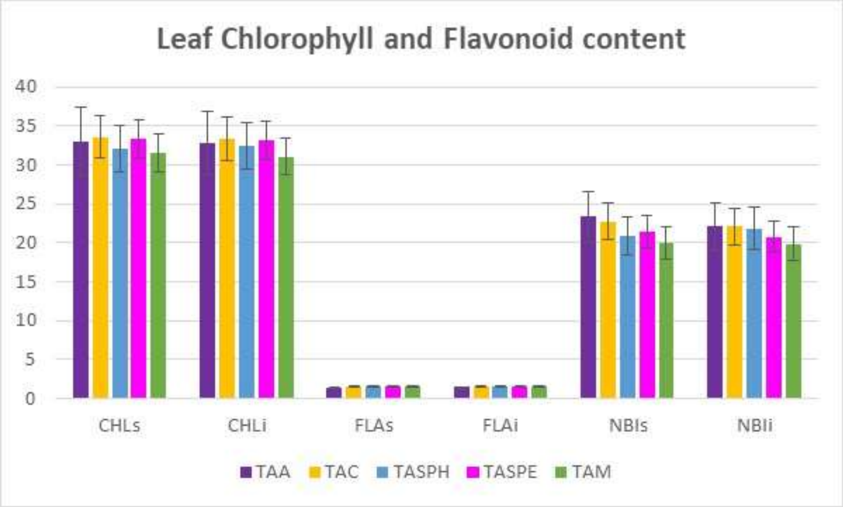

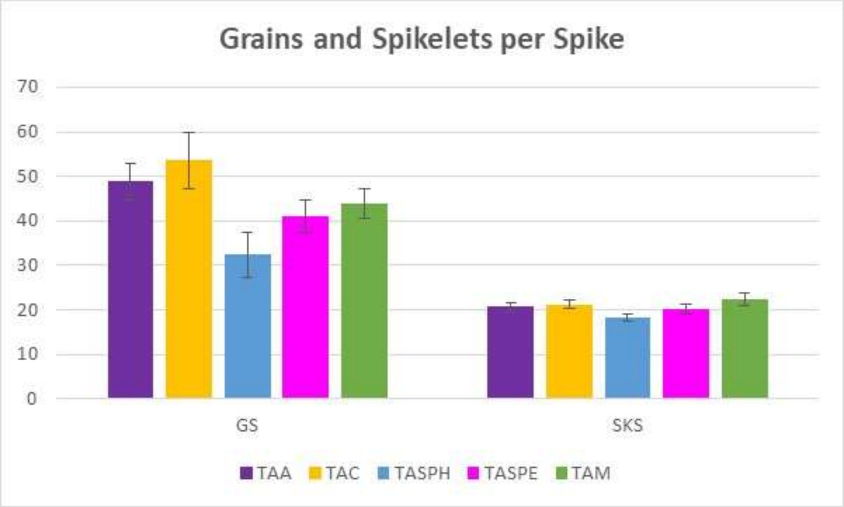

Data-points
GNS: 291
SNS: 291

Phenotyping Fiorenzuola 2018 (boxplots)

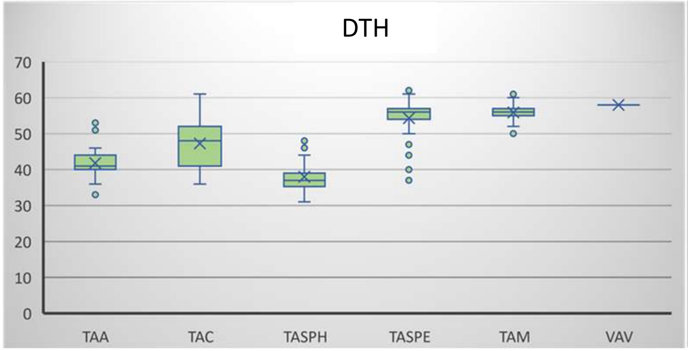

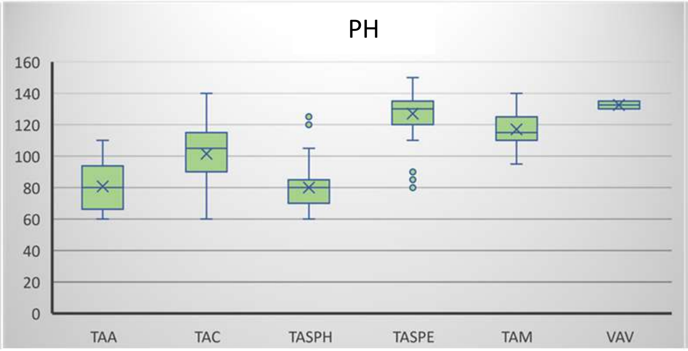

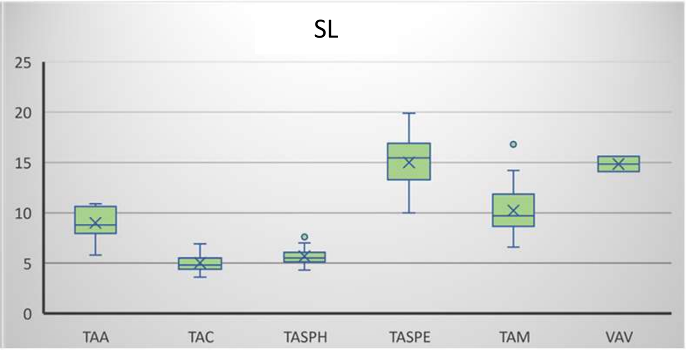

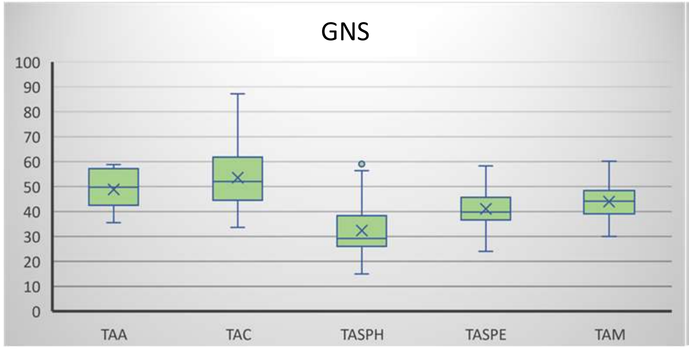

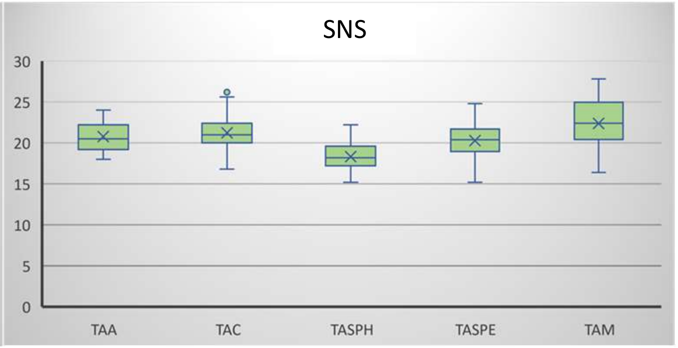

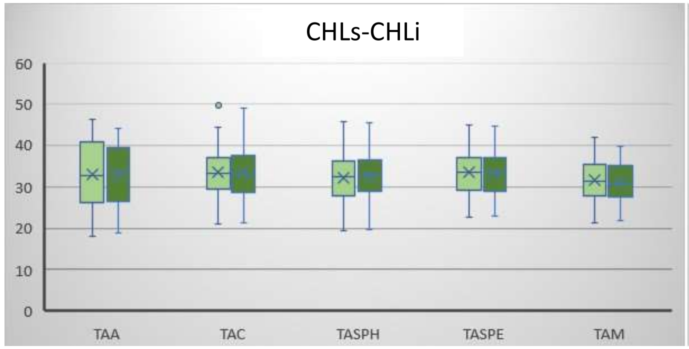

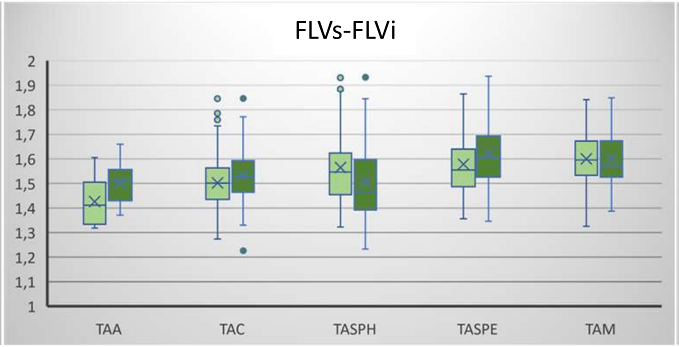

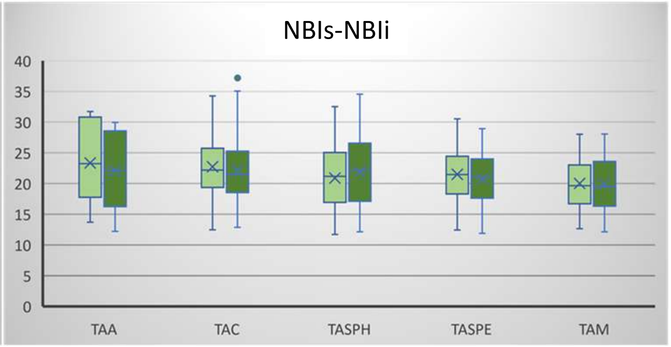

Phenotyping Fiorenzuola 2018 (boxplots)

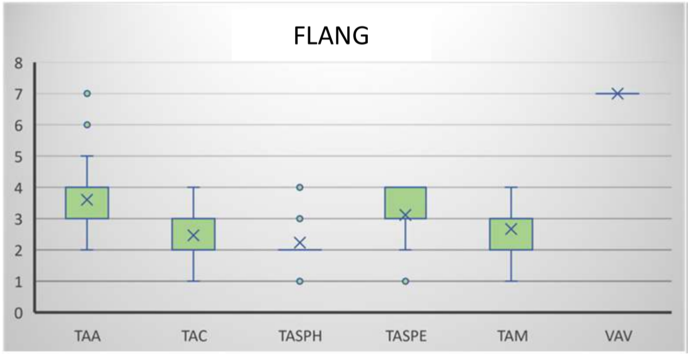

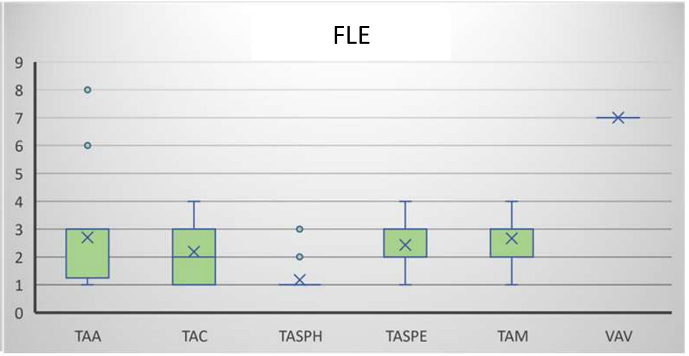

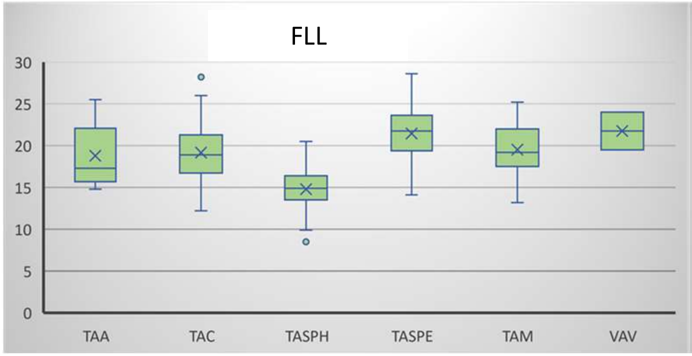

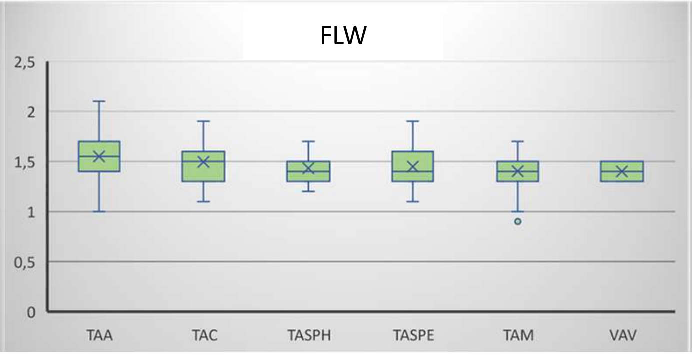

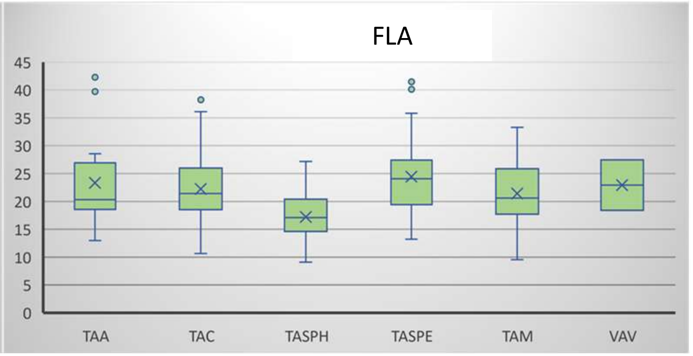

Phenotyping Fiorenzuola 2019 (191 genotypes, 3 rep)

Data-points
DTH: 569
PH: 568

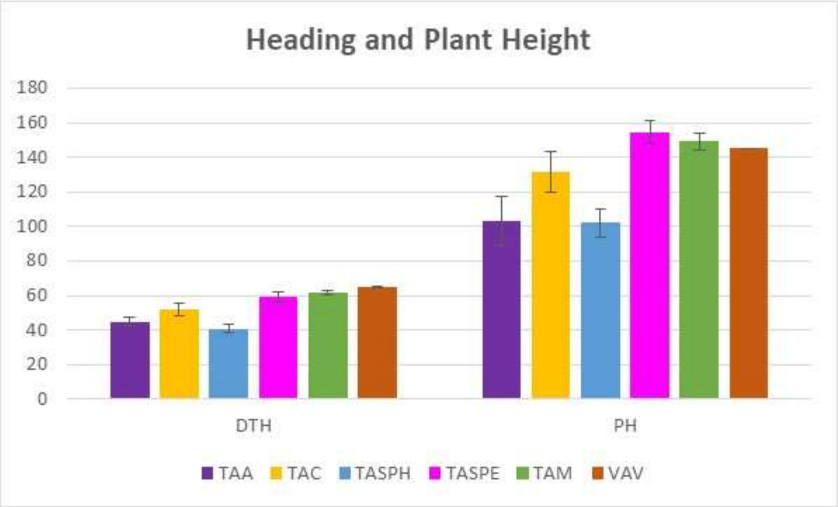

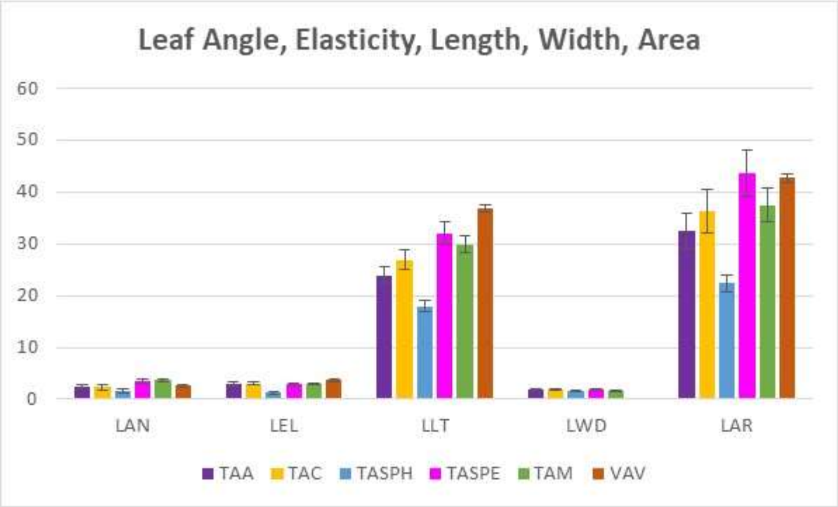

Data-points
FLANG: 567
FLE: 569
FLL: 567
FLW: 567
FLA: 567

Data-points
CHL: 568
FLV: 568
NBI: 568

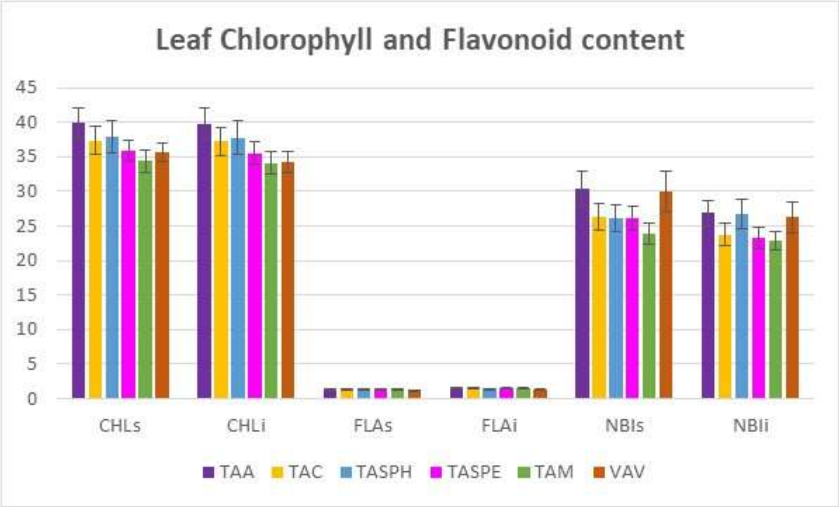

Phenotyping Fiorenzuola 2019 (191 genotypes, 3 rep)

Data-points
SL: 270
SW: 188
SD^: 188

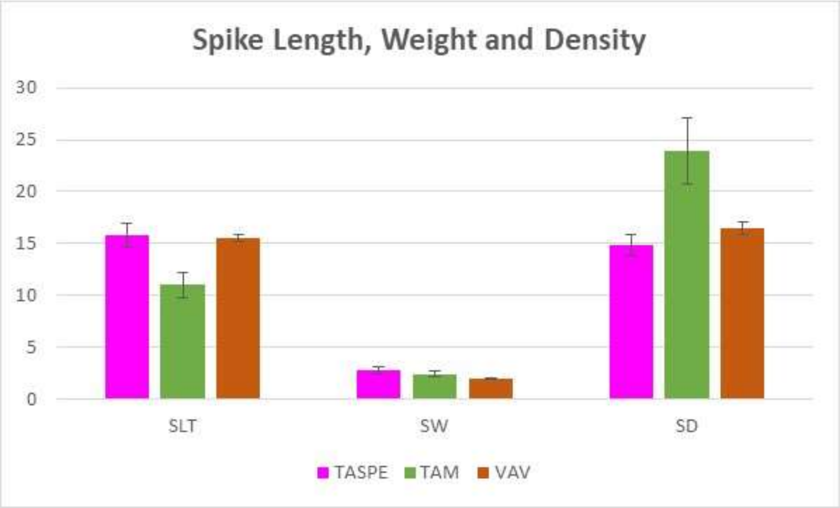

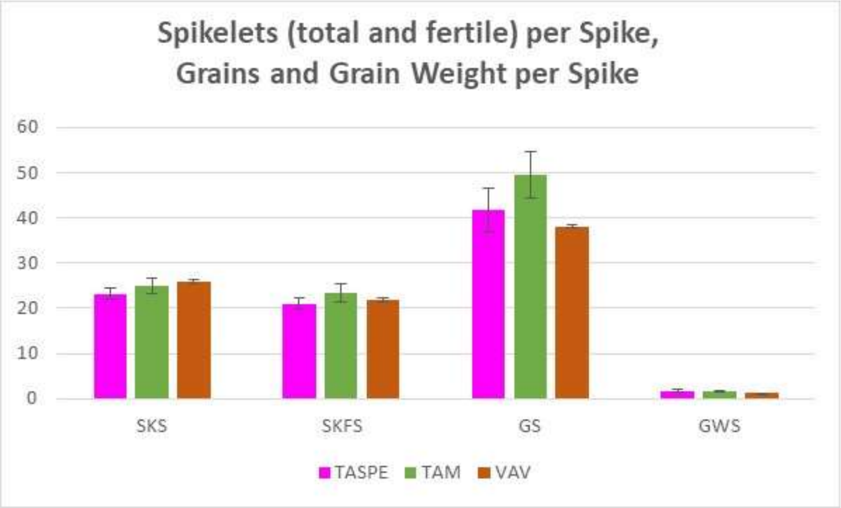

Data-points
SNS: 188
FSNS: 188
GNS: 188
GWS: 188

Phenotyping Fiorenzuola 2019 (boxplots)

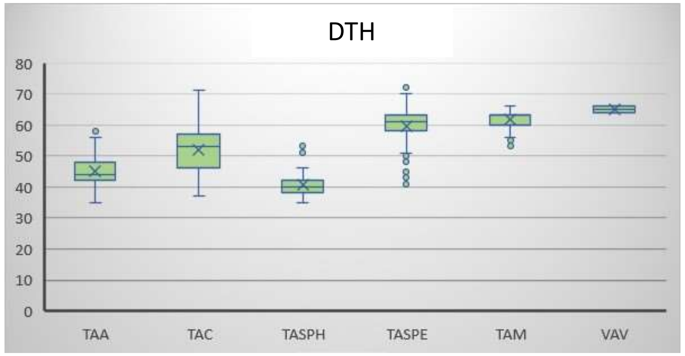

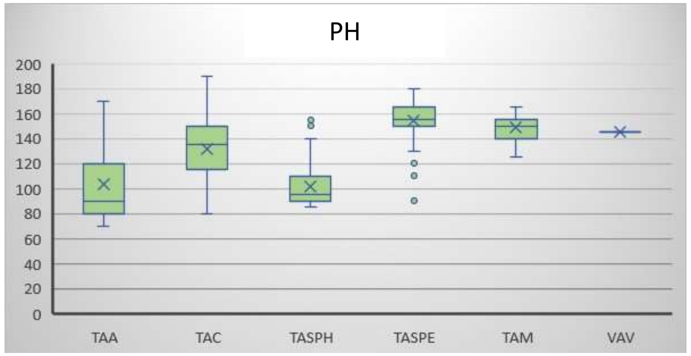

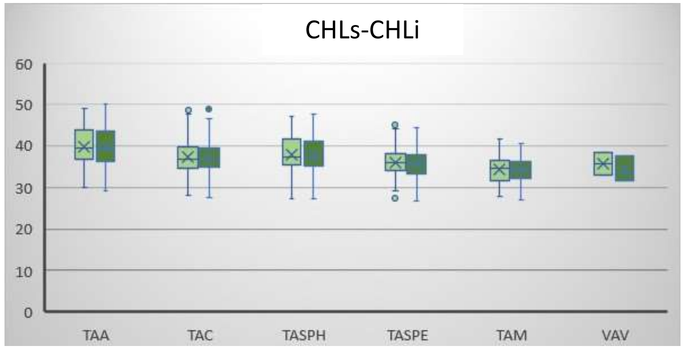

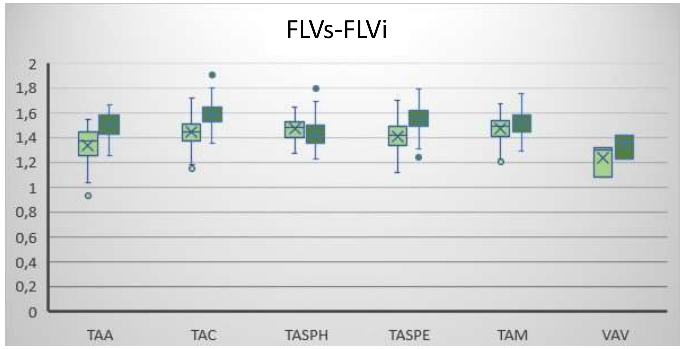

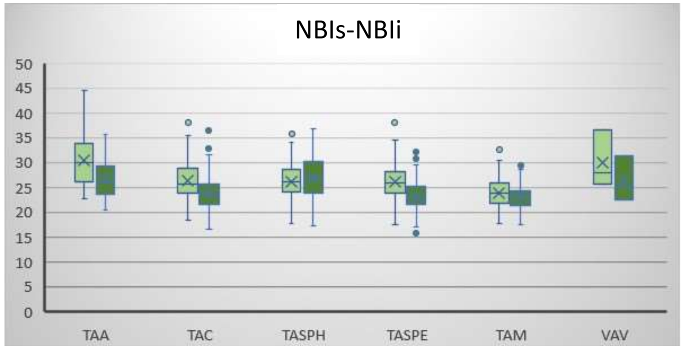

Phenotyping Fiorenzuola 2019 (boxplots)

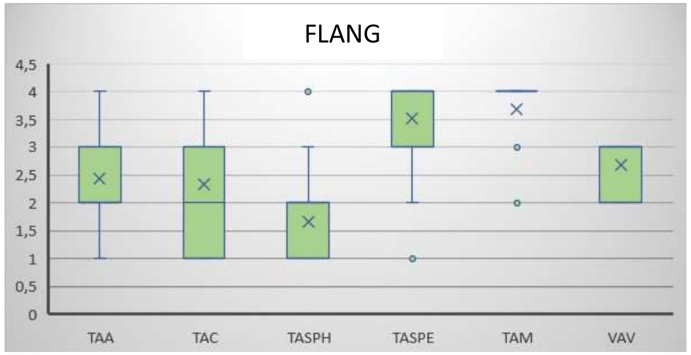

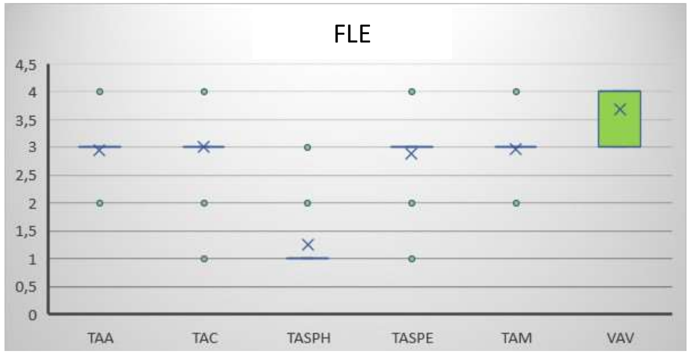

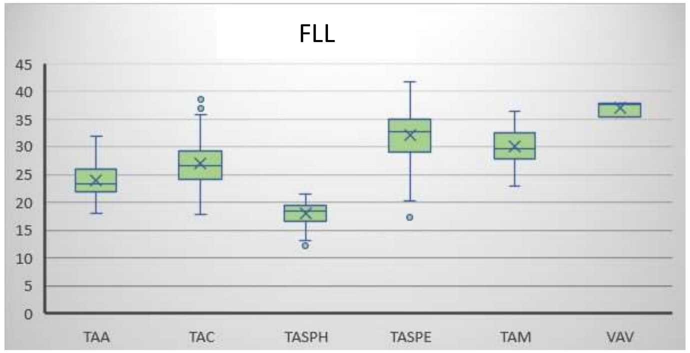

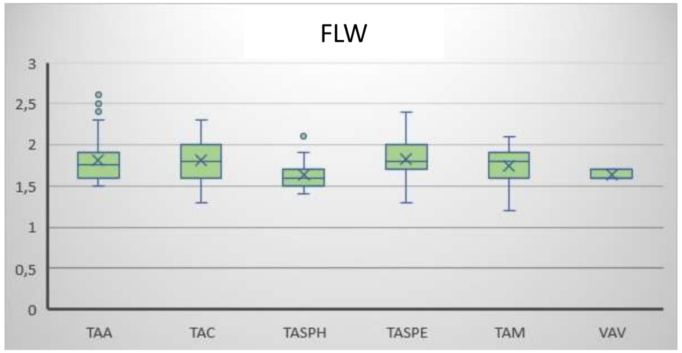

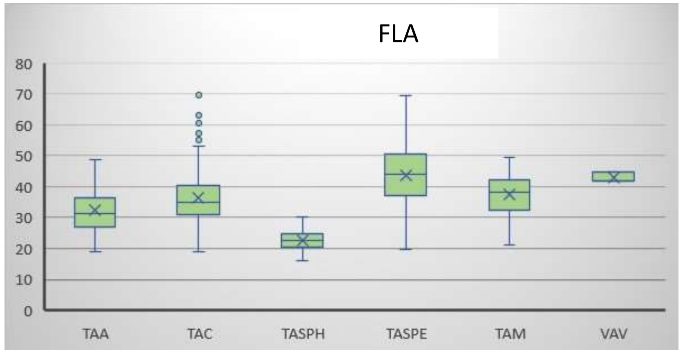

Phenotyping Fiorenzuola 2019 (boxplots)

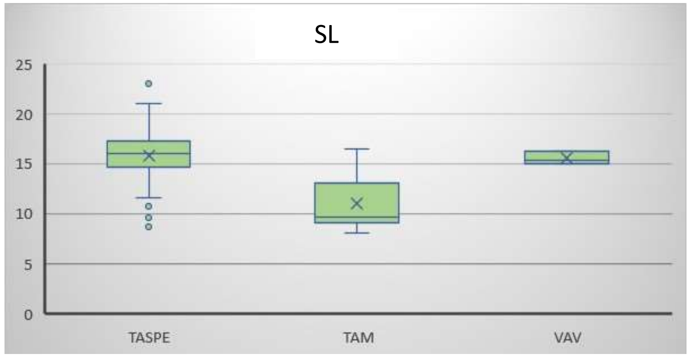

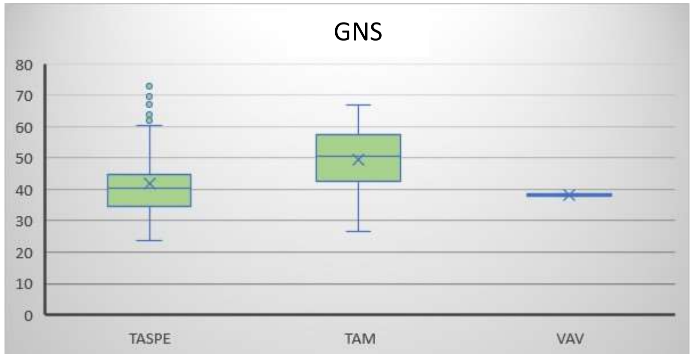

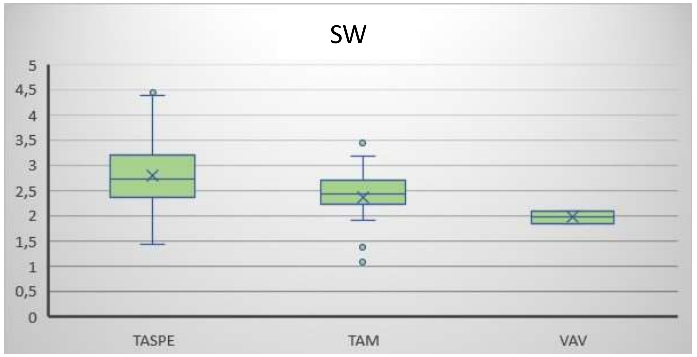

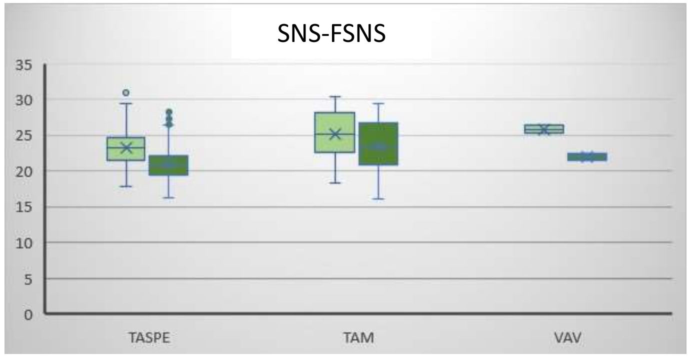

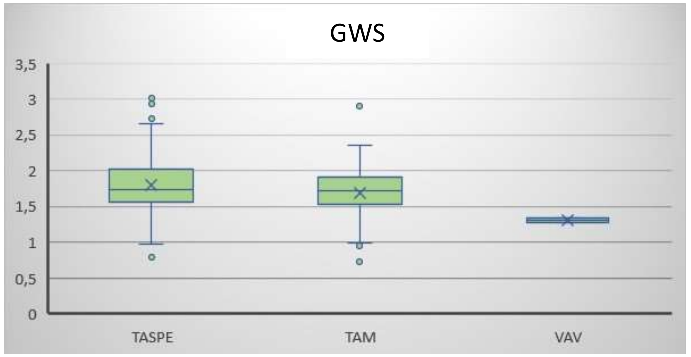

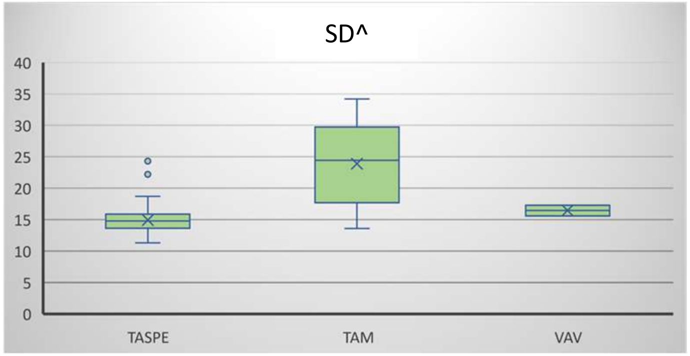

Phenotyping Fiorenzuola 2021 (190 genotypes, 2 rep)

Data-points
DTH: 379
PH: 378

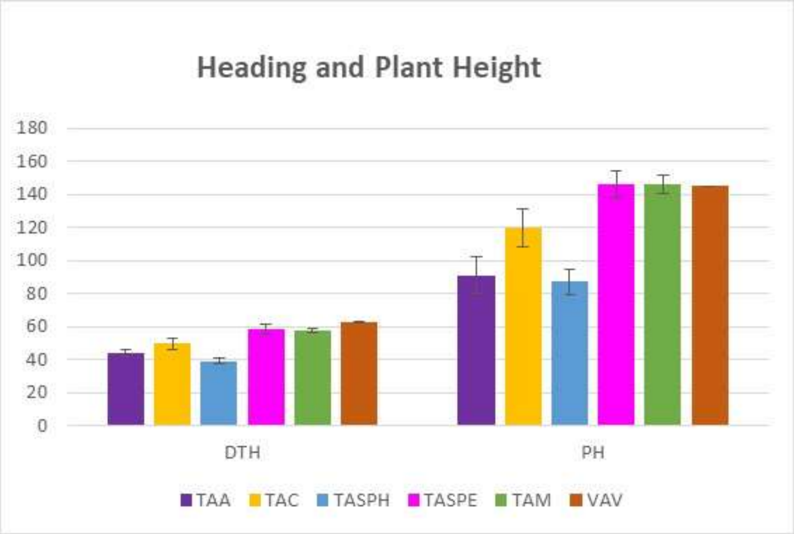

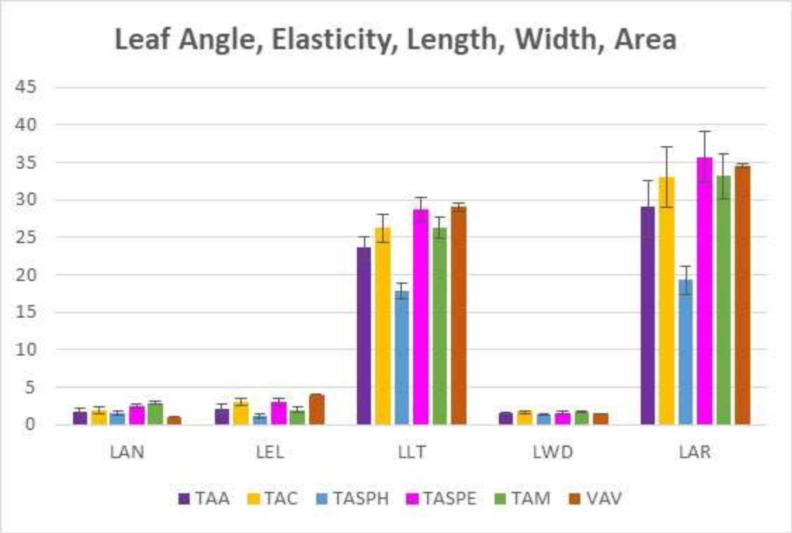

Data-points
FLANG: 379
FLE: 378
FLL: 377
FLW: 377
FLA: 377

Data-points
CHL: 379
FLV: 379
NBI: 379

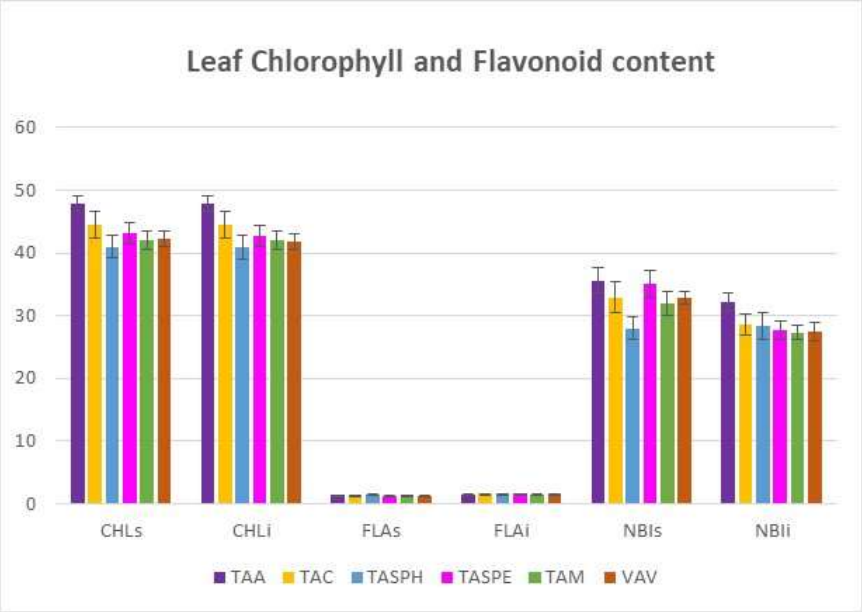

Phenotyping Fiorenzuola 2021 (190 genotypes, 2 rep)

Data-points
SL: 379
SW: 372
SD^: 371

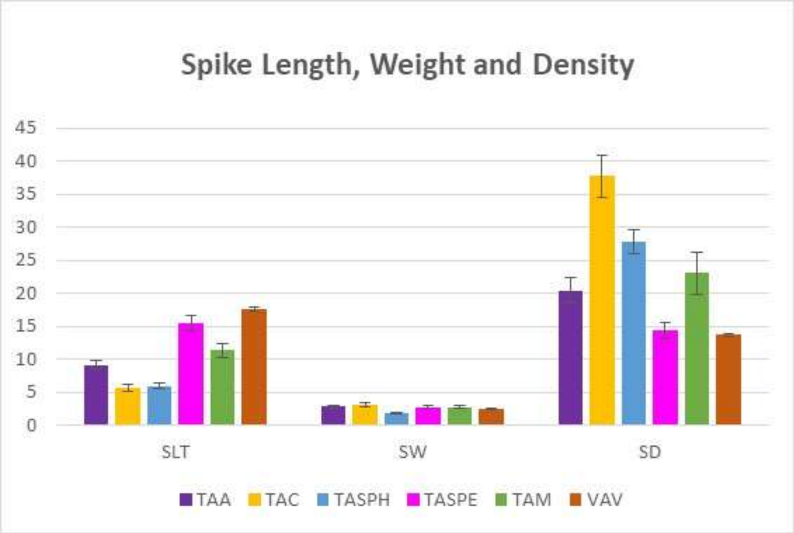

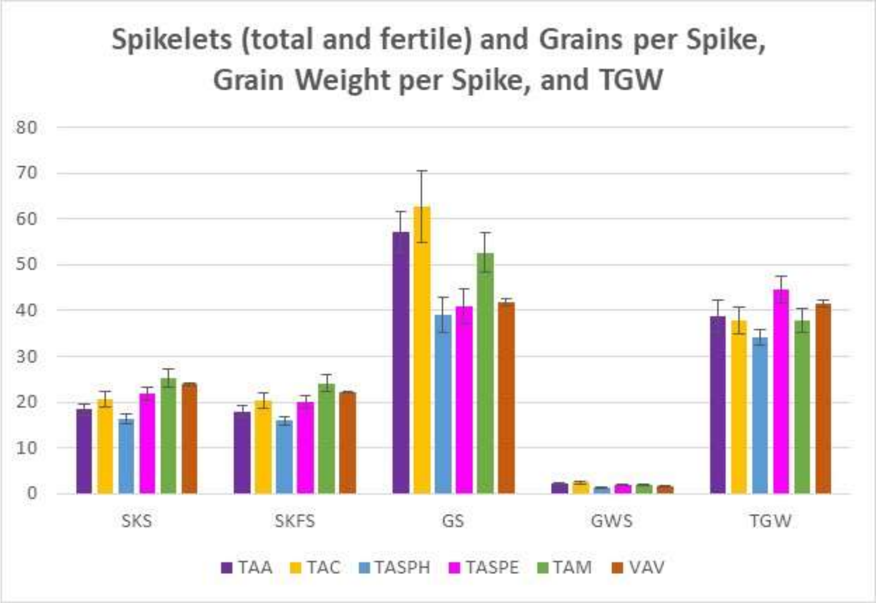

Data-points
SNS: 373
FSNS: 373
GNS: 371
GWS: 371
TGW: 370

Phenotyping Fiorenzuola 2021 (boxplots)

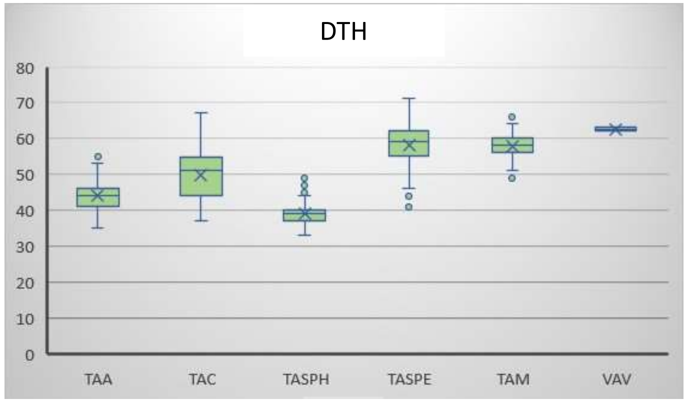

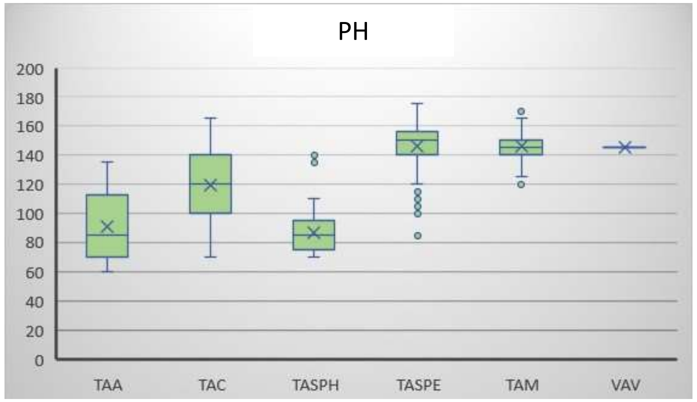

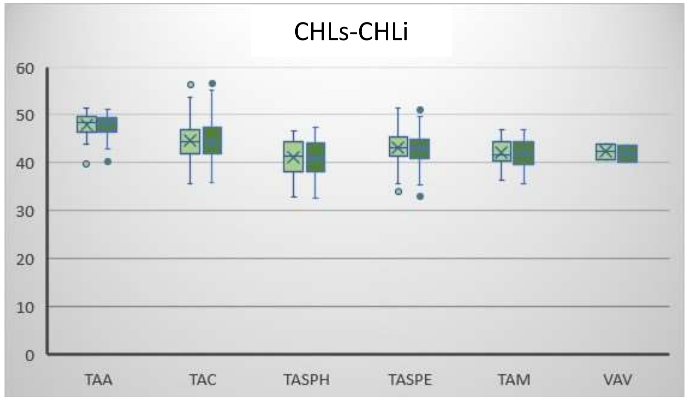

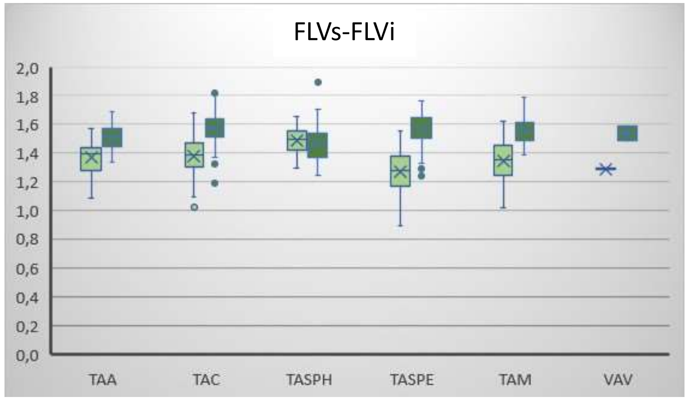

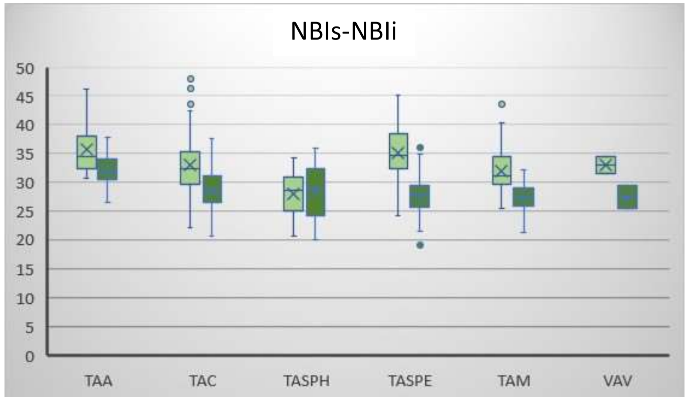

Phenotyping Fiorenzuola 2021 (boxplots)

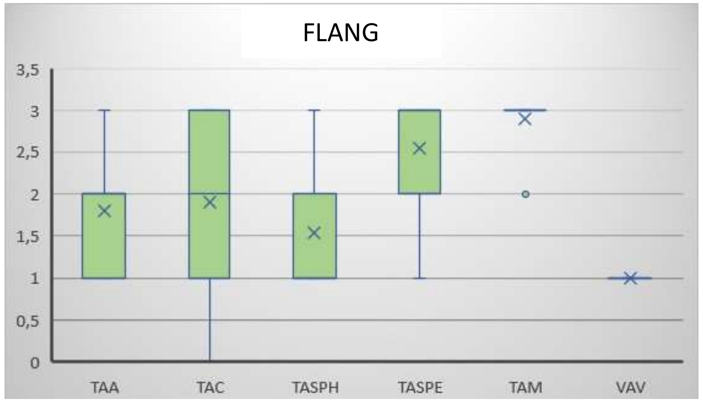

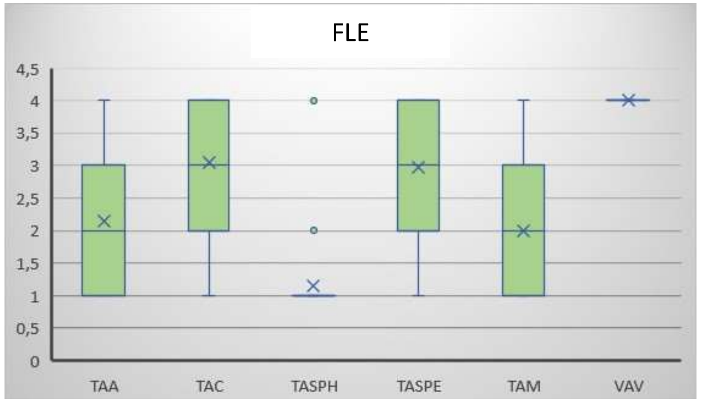

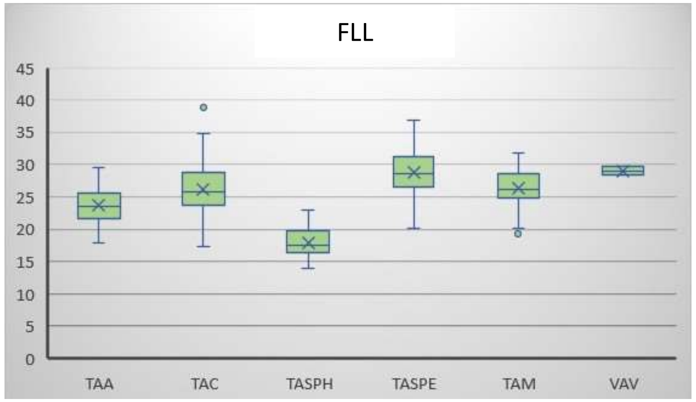

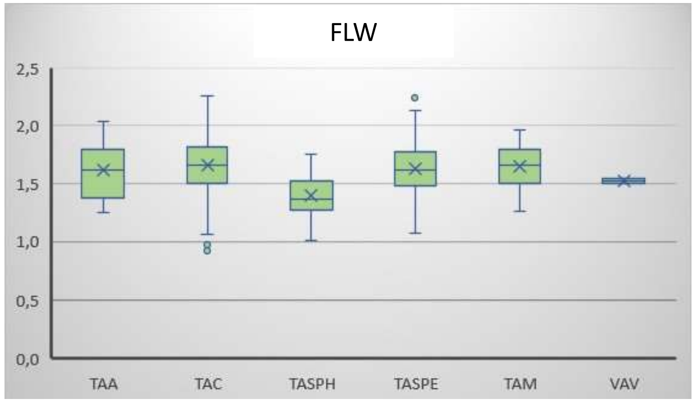

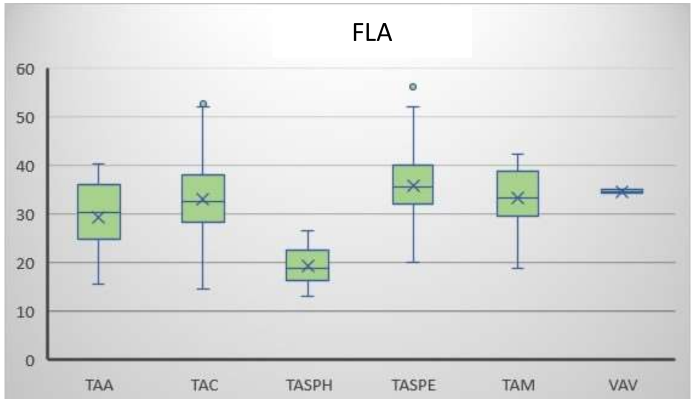

Phenotyping Fiorenzuola 2021 (boxplots)

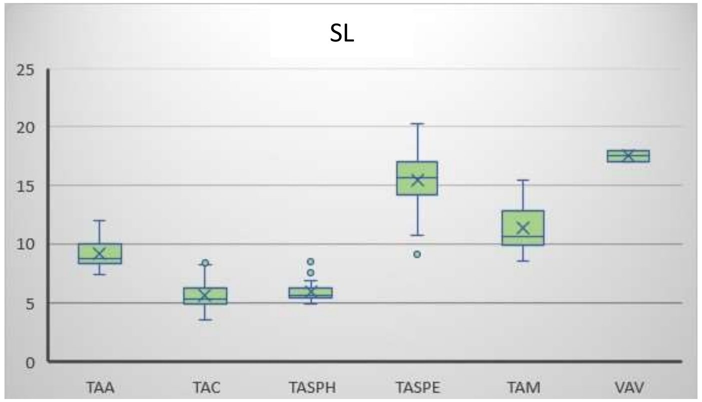

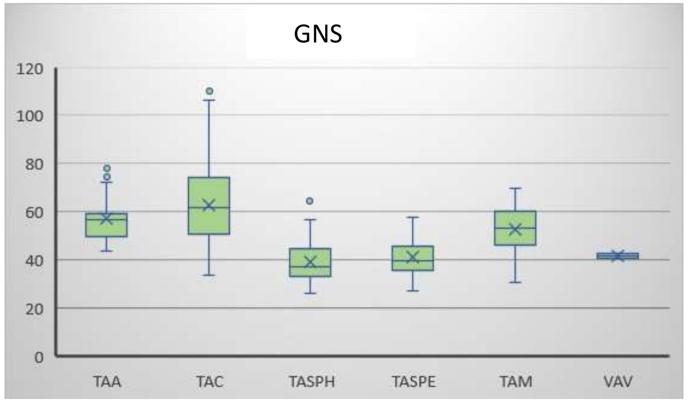

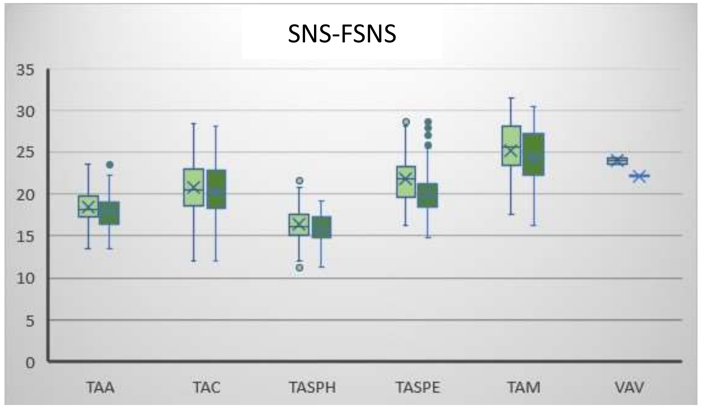

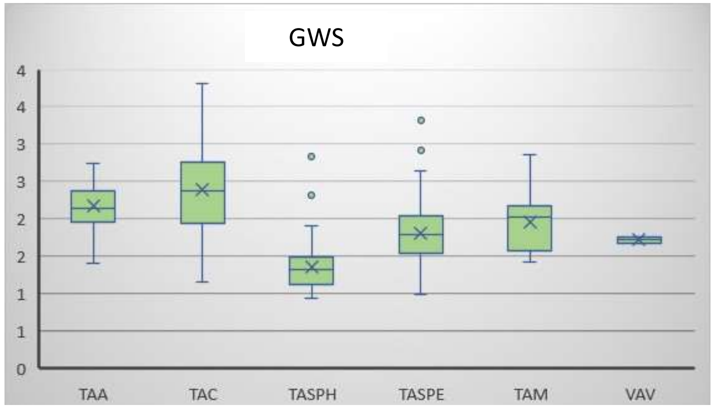

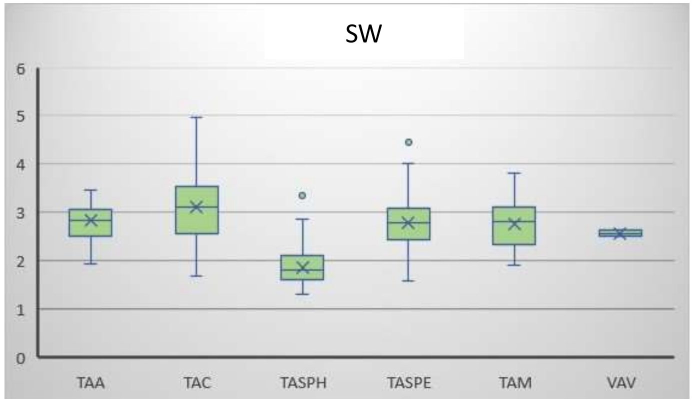

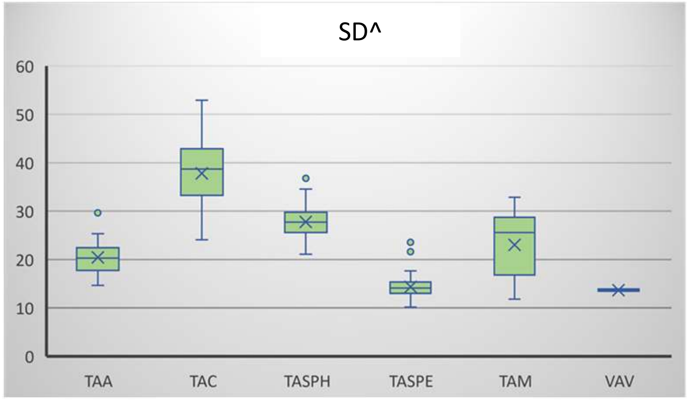

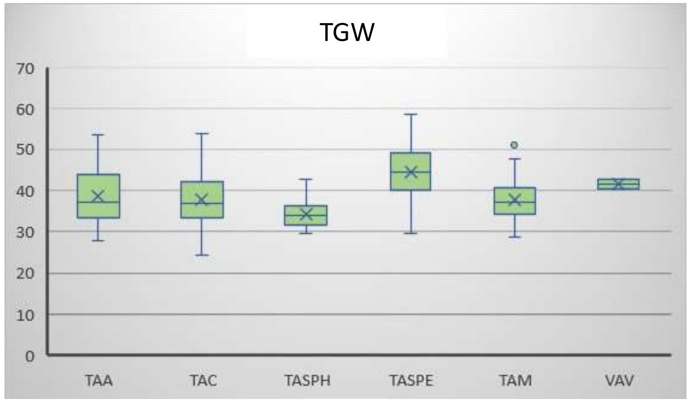

Phenotyping Fiorenzuola 2022 (190 genotypes, 2 rep)

Data-points
DTH: 380
PH: 380

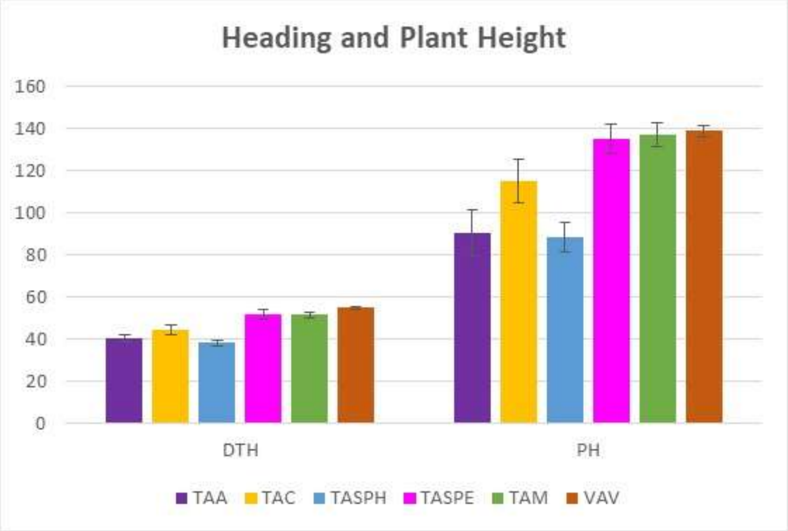

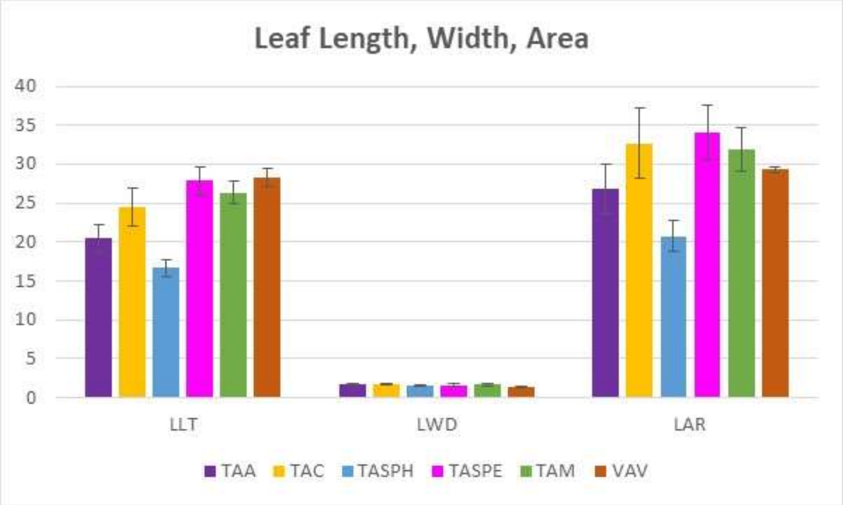

Data-points
FLL: 380
FLW: 380
FLA: 380

Data-points
CHL: 380
FLV: 380
NBI: 380

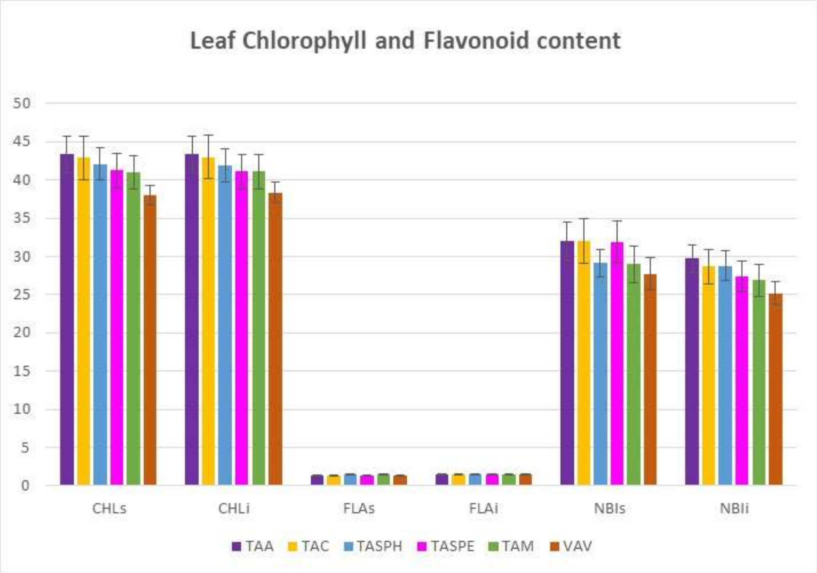

Phenotyping Fiorenzuola 2022 (190 genotypes, 2 rep)

Data-points
SL: 373
SW: 373
SD^: 370

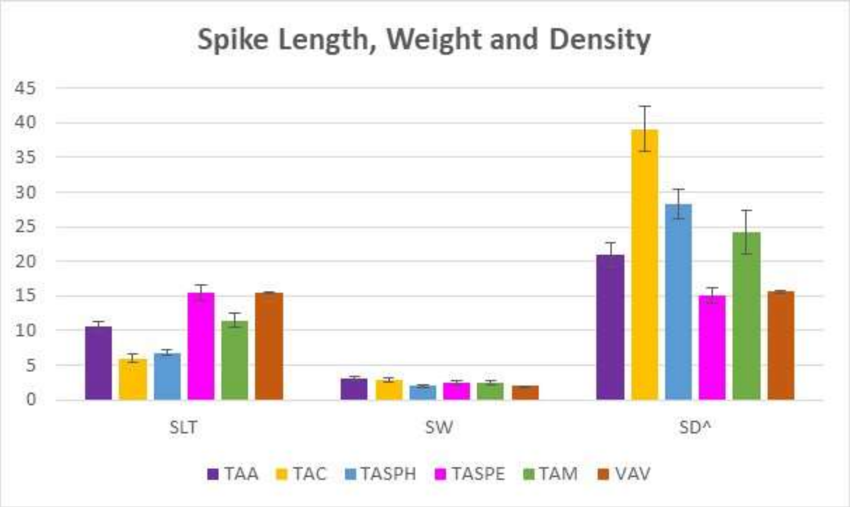

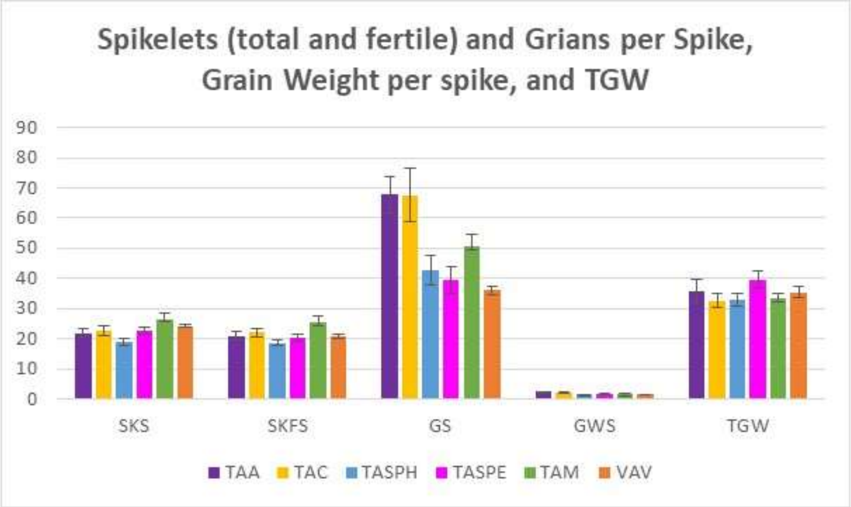

Data-points
SNS: 371
FSNS: 371
GNS: 373
GWS: 373
TGW: 379

Phenotyping Fiorenzuola 2022 (boxplots)

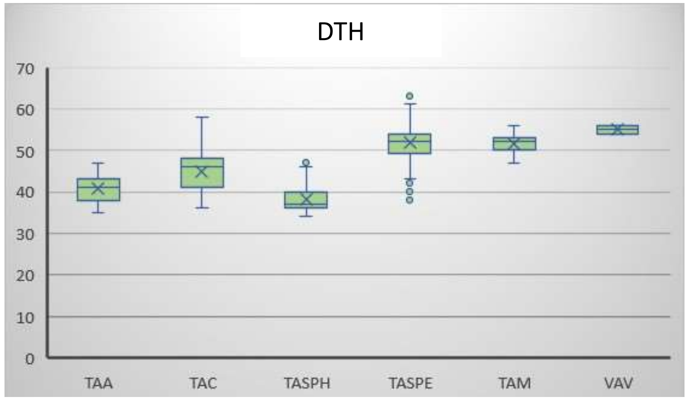

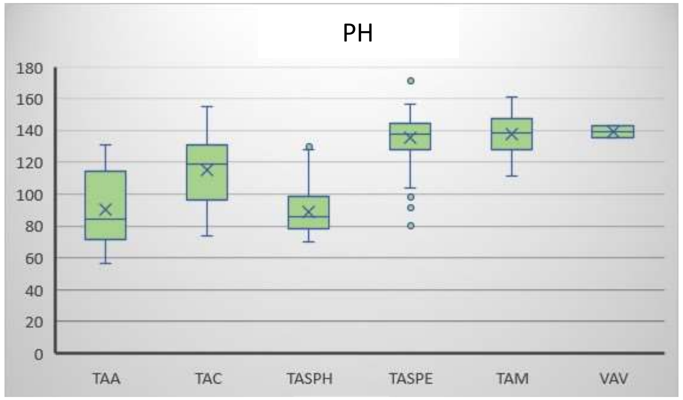

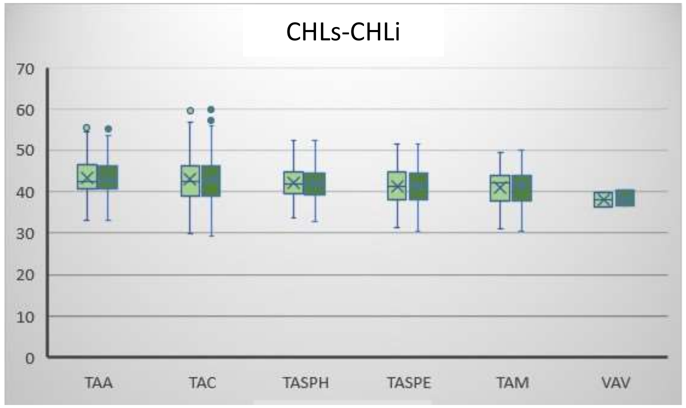

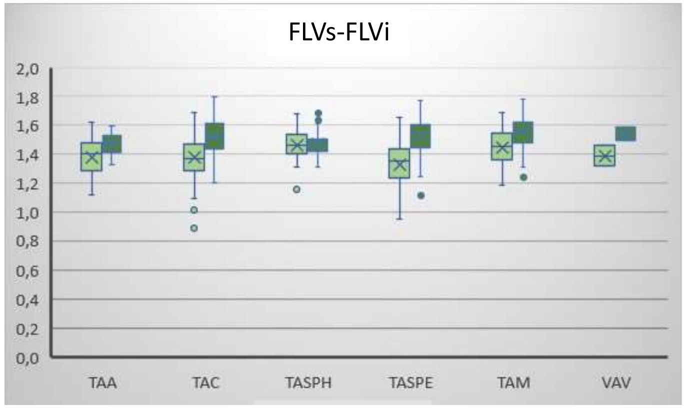

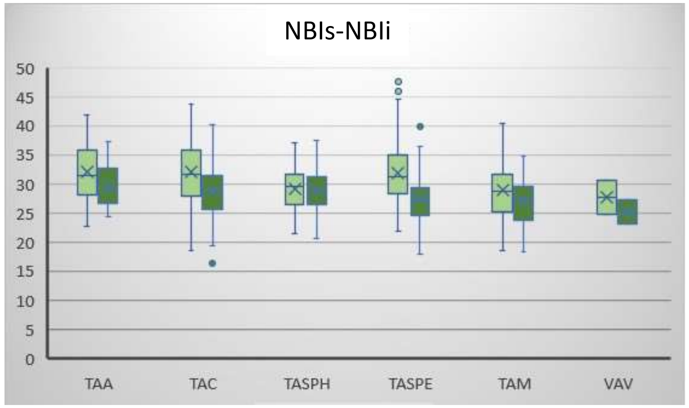

Phenotyping Fiorenzuola 2022 (boxplots)

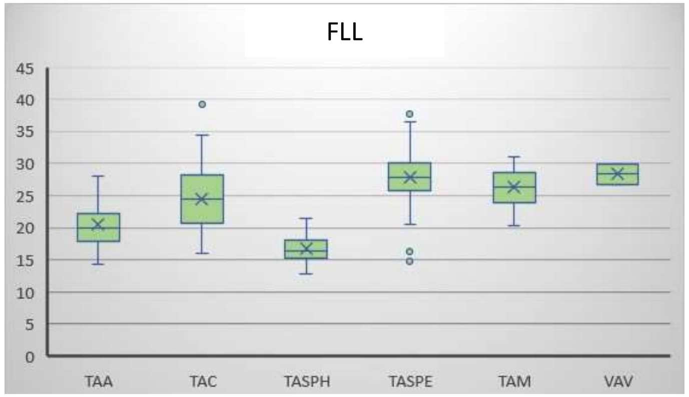

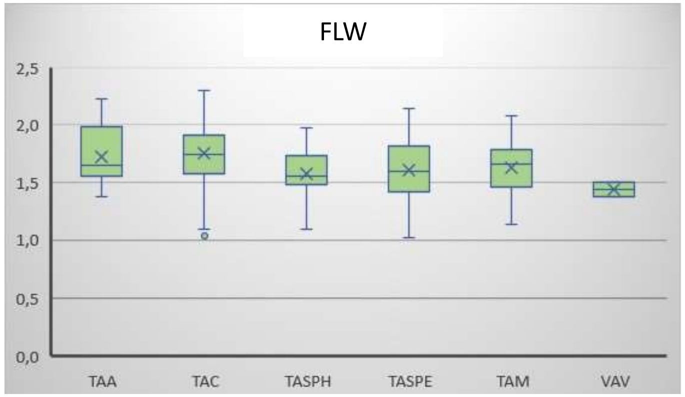

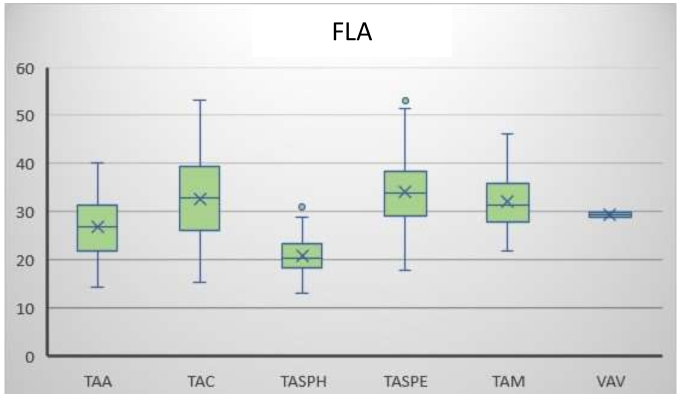

Phenotyping Fiorenzuola 2022 (boxplots)

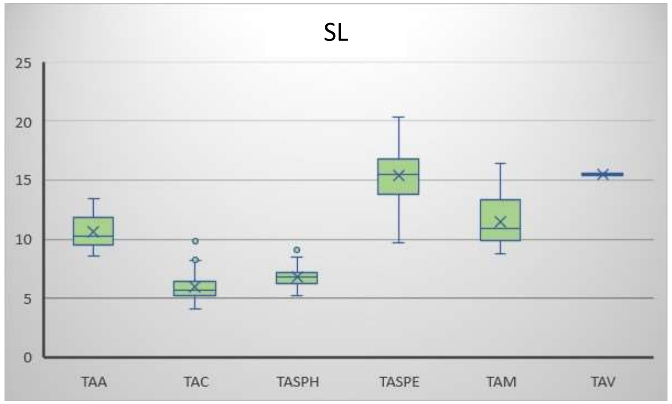

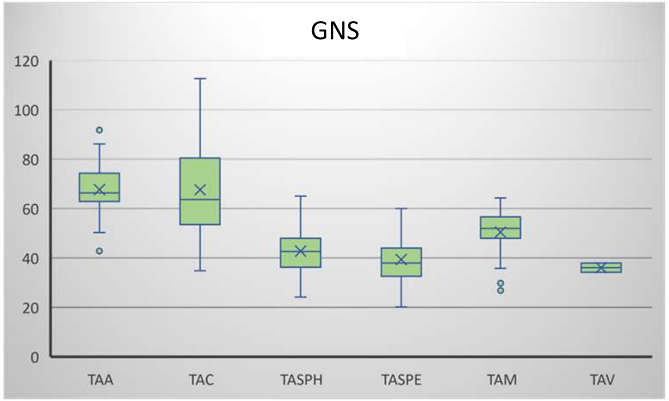

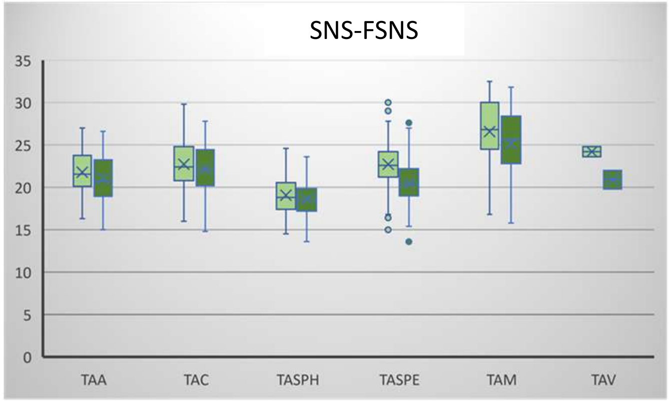

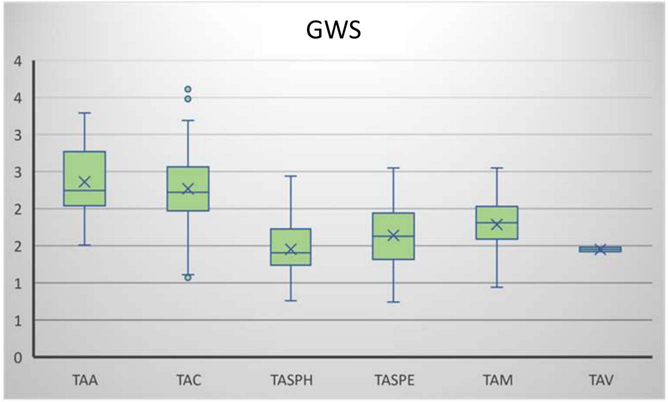

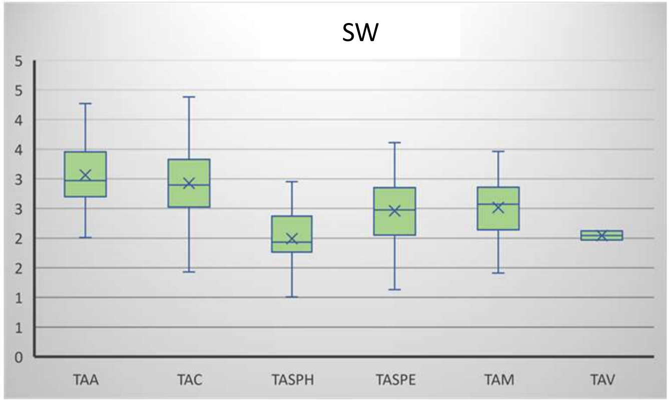

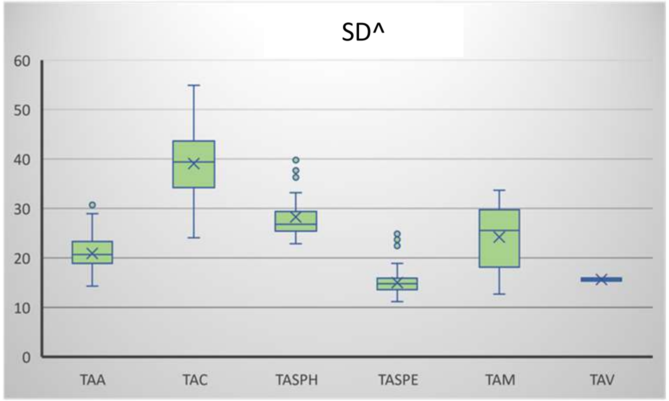

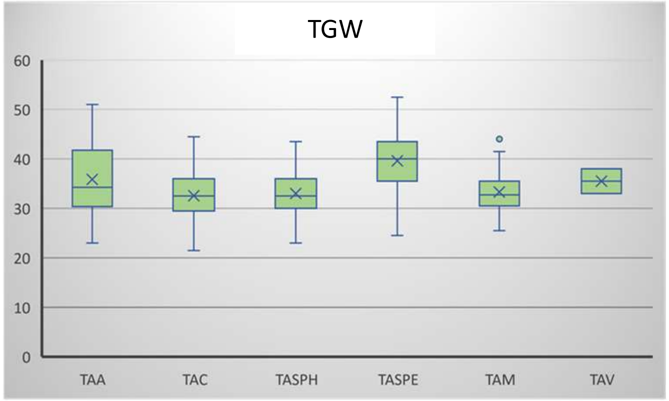

Supplement: Supplementary file 1 [file Image1.pdf]
