# Supplementary figures and images for: Ancient diversity of Triticum aestivum subspecies as source of novel loci for bread wheat improvement
Source: Front Plant Sci. 2025 Apr 9;16:1536991. doi: 10.3389/fpls.2025.1536991 (PMC12014548; doi:10.3389/fpls.2025.1536991)

# Delta K

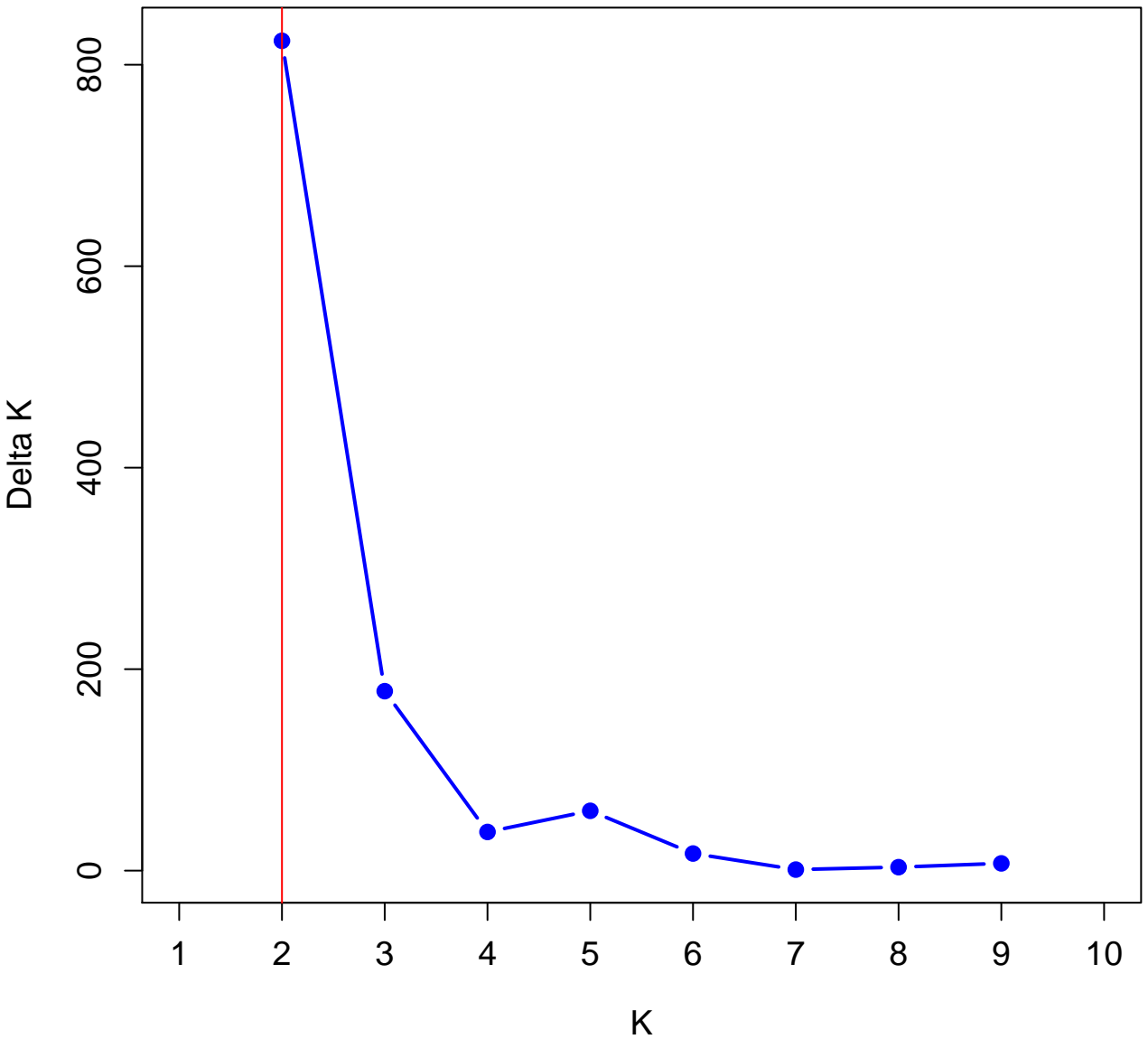

Supplement: Supplementary file 2 [file Image2.pdf]

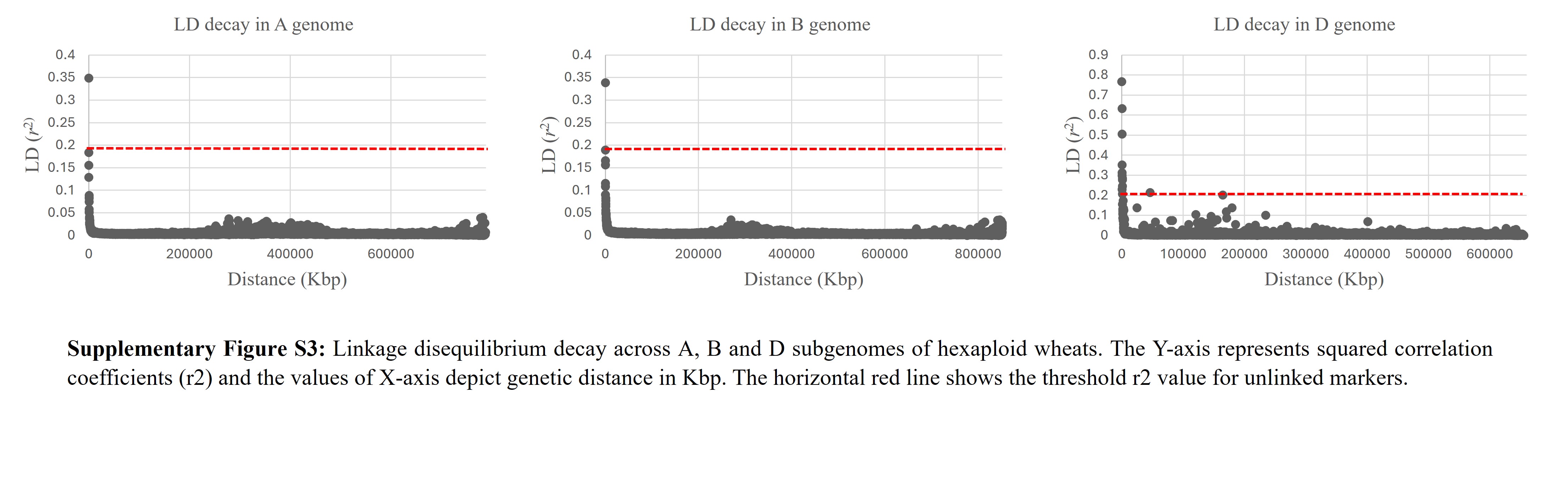

Supplement: Supplementary file 3 [file Image3.jpg]
