# Supplementary material for: Ancient diversity of Triticum aestivum subspecies as source of novel loci for bread wheat improvement
Source: Front Plant Sci. 2025 Apr 9;16:1536991. doi: 10.3389/fpls.2025.1536991 (PMC12014548; doi:10.3389/fpls.2025.1536991)
Supplement: Supplementary file 4 [file Image4.pdf]

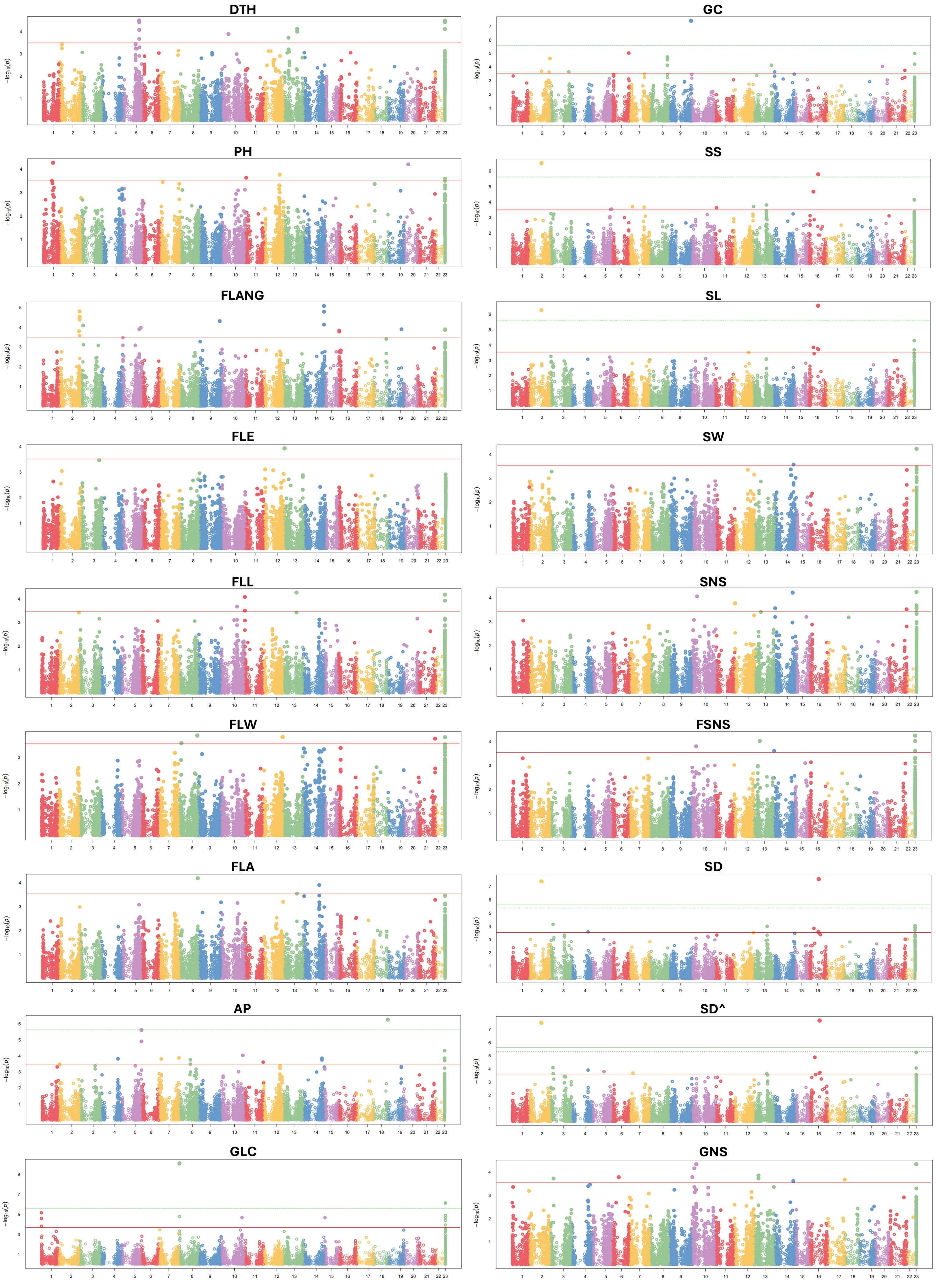
DTH
GC
PH
SS
FLANG
SL
FLE
SW
FLL
SNS
FLW
FSNS
FLA
SD
AP
SD^
GLC
GNS
$-\log_{10}(p)$
1 2 3 4 5 6 7 8 9 10 11 12 13 14 15 16 17 18 19 20 21 22 23

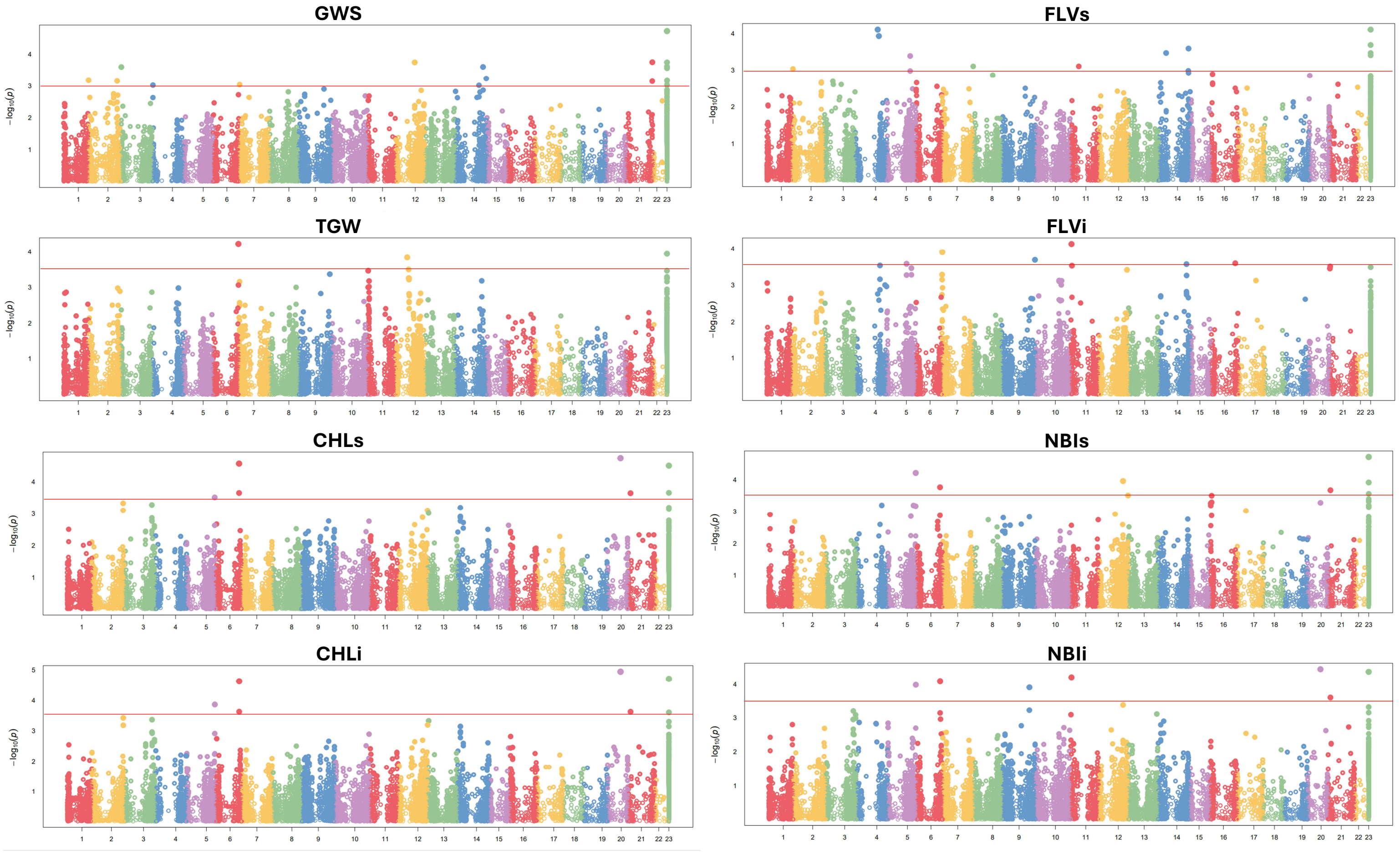

Supplementary Figure S4: Manhattan plots for all analysed traits. The red line represents the significance threshold chosen in this study (−log10 (*P*-value) ≥ 3.5) to define the MTAs, while the green line represents the significance threshold of Bonferroni.
