# Supplementary material for: Ancient diversity of Triticum aestivum subspecies as source of novel loci for bread wheat improvement
Source: Front Plant Sci. 2025 Apr 9;16:1536991. doi: 10.3389/fpls.2025.1536991 (PMC12014548; doi:10.3389/fpls.2025.1536991)
Supplement: Supplementary file 14 [file DataSheet1.pdf]

Supplementary Table and Figure legend

Supplementary Table S1: List of 190 genotypes along with all relevant information

Supplementary Table S2: Phenotyping legend

Supplementary Table S3: All the information on markers and alleles of the known genes tested on *Ta*-ssp panel

Supplementary Table S4: A) Means, median, minimum, maximum, and quartiles for all traits in each year; B) Minimum, maximum, and mean values for each subspecies in each year; C) Mean values of the 21 non-categorical traits for each subspecies, averaged over at least two years

Supplementary Table S5: Coefficients of pairwise Pearson's correlations among the agronomic traits calculated on BLUPs

Supplementary Table S6: A) Allelic patterns at known genes across the *Ta*-ssp panel; B) Allelic distribution at known genes in different subspecies (*vavilovii* is not included)

Supplementary Table S7: A) Physical locations on the Chinese Spring RefSeq v2.1; B) Molecular marker distribution (14,640 SNPs) in the A, B, D, and whole genomes

Supplementary Table S8: Output of GWAS results including the MTAs at −log10 $P \geq 3.5$ for all traits analysed

Supplementary Table S9: HC genes located in the MTA regions associated to multiple traits (peak marker position ± LD for specific for each chromosome) and the relative functional annotation base on IWGSC RefSeq v2.1

Supplementary Figure S1: Distribution of the traits intra subspecies: barplots and boxplots for the four years

Supplementary Figure S2: Delta K graph obtained with Evanno method in StructureSelector https://lmme.ac.cn/StructureSelector/tmp/1734358609.out/Results.html

Supplementary Figure S3: Linkage disequilibrium decay across A, B and D subgenomes of hexaploid wheats, and a focus along a distance of 10000bp. The Y-axis represents squared correlation coefficients (r^2) and the values of X-axis depict genetic distance in Kbp

Supplementary Figure S4: Manhattan plots for all analysed traits. The red line represents the significance threshold chosen in this study ($-\log_{10}$ (P-value) ≥ 3.5) to define the MTAs, while the green line represents the significance threshold of Bonferroni.
