# Supplementary material for: Ancient diversity of Triticum aestivum subspecies as source of novel loci for bread wheat improvement
Source: Front Plant Sci. 2025 Apr 9;16:1536991. doi: 10.3389/fpls.2025.1536991 (PMC12014548; doi:10.3389/fpls.2025.1536991)
Supplement: Supplementary file 15 [file SupplementaryFile1.pdf]

References cited only in the Supplementary Material

Beales, J., Turner, A., Griffiths, S., Snape, J. W., Laurie, D. A. (2007). A pseudo-response regulator is misexpressed in the photoperiod insensitive *Ppd-D1a* mutant of wheat (*Triticum aestivum* L.). *Theor. Appl. Genet.* 115, 721-33. doi: 10.1007/s00122-007-0603-4

Ellis, M.H., Spielmeyer, W., Gale, K. R., Rebetzke, G. J., Richards, R. A. (2002). “Perfect” markers for the Rht-B1b and Rht-D1b dwarfing genes in wheat. *Theor. Appl. Genet.* 105, 1038–1042. DOI: 10.1007/s00122-002-1048-4

Hou, J., Jiang, Q., Hao, C., Wang, Y., Zhang, H., Zhang, X. (2014). Global selection on sucrose synthase haplotypes during a century of wheat breeding. *Plant Physiol.* 164(4),1918-29. doi: 10.1104/pp.113.232454

Jiang, Q., Hou, J., Hao, C., Wang, L., Ge, H., Dong, Y., et al. (2011). The wheat (T. aestivum) sucrose synthase 2 gene (TaSus2) active in endosperm development is associated with yield traits. *Funct. Integr. Genomics* 11, 49-61. doi: 10.1007/s10142-010-0188-x.

Jiang, Y., Jiang, Q., Hao, C., Hou, J., Wang, L., Zhang, H., et al. (2015). A yield-associated gene TaCWI, in wheat: its function, selection and evolution in global breeding revealed by haplotype analysis. *Theor. Appl. Genet.* 128, 131-43. doi: 10.1007/s00122-014-2417-5

Ma, D. Y., Yan, J., He, Z. H., Wu, L., Xia, X.C. (2012). Characterization of a cell wall invertase gene TaCwi-A1 on common wheat chromosome 2A and development of functional markers. *Mol Breeding* 29, 43–52. https://doi.org/10.1007/s11032-010-9524-z

Mohammadi, M., Mehrazar, E., Izadi-Darbandi, A., Najafian, G. (2013). Genotype Diversity of Puroindoline Genes (*Pina-D1* and *Pinb-D1*) in Bread Wheat Cultivars Developed in Iran and CIMMYT. *J. Crop Improv.* 27(4), 361-375. DOI: 10.1080/15427528.2013.775988

Su, Z. Q., Hao, C. Y., Wang, L. F., Dong, Y. C., Zhang, X. Y. (2011). Identification and development of a functional marker of TaGW2 associated with grain weight in bread wheat (*Triticum aestivum* L.). *Theor. Appl. Genet.* 122, 211–223. doi:10.1007/s00122-010-1437-z

Uauy, C., Distelfeld, A., Fahima, T., Blechl, A., Dubcovsky, J. (2006). A NAC Gene Regulating Senescence Improves Grain Protein, Zinc, and Iron Content in Wheat. *Science* 314, 1298-1301. DOI: 10.1126/science.1133649

Zanke, C., Ling, J., Plieske, J., Kollers, S., Ebmeyer, E., Korzun, V., et al. (2015). Analysis of main effect QTL for thousand grain weight in European winter wheat (*Triticum aestivum* L.) by genome-wide association mapping. *Front. Plant Sc*. 6, 1-14. https://doi.org/10.3389/fpls.2015.00644

Zhang, L., Zhao, Y-L., Gao, L-F., Zhao, G-Y., Zhou, R-H., Zhang, B-S., et al. (2012). TaCKX6-D1, the ortholog of rice OsCKX2, is associated with grain weight in hexaploid wheat. *New Phytol.* 195, 574–584. https://doi.org/10.1111/j.1469-8137.2012.04194.x
